# Targeting oncogenic *TERT* promoter variants by allele-specific epigenome editing

**DOI:** 10.1186/s13148-023-01599-2

**Published:** 2023-11-22

**Authors:** Alexandra G. Kouroukli, Nivethika Rajaram, Pavel Bashtrykov, Helene Kretzmer, Reiner Siebert, Albert Jeltsch, Susanne Bens

**Affiliations:** 1 Institute of Human Genetics, Ulm University and Ulm University Medical Center, Albert-Einstein-Allee 11, 89081 Ulm, Germany; 2 Department of Biochemistry, Institute of Biochemistry and Technical Biochemistry, University of Stuttgart, Allmandring 31, 70569 Stuttgart, Germany; 3 Computational Genomics, Department of Genome Regulation, Max Planck Institute for Molecular Genetics, Berlin, Germany

**Keywords:** Allele-specific epigenome editing (ASEE), Telomerase reverse transcriptase, *TERT*, Single-nucleotide variants, DNA methylation, Cancer

## Abstract

**Background:**

Activation of dominant oncogenes by small or structural genomic alterations is a common driver mechanism in many cancers. Silencing of such dominantly activated oncogenic alleles, thus, is a promising strategy to treat cancer. Recently, allele-specific epigenome editing (ASEE) has been described as a means to reduce transcription of genes in an allele-specific manner. In cancer, specificity to an oncogenic allele can be reached by either targeting directly a pathogenic single-nucleotide variant or a polymorphic single-nucleotide variant linked to the oncogenic allele. To investigate the potential of ASEE in cancer, we here explored this approach by targeting variants at the *TERT* promoter region. The *TERT* promoter region has been described as one of the most frequently mutated non-coding cancer drivers.

**Results:**

Sequencing of the *TERT* promoter in cancer cell lines showed 53% (41/77) to contain at least one heterozygous sequence variant allowing allele distinction. We chose the hepatoblastoma cell line Hep-G2 and the lung cancer cell line A-549 for this proof-of-principle study, as they contained two different kinds of variants, namely the activating mutation C228T in the *TERT* core promoter and the common SNP rs2853669 in the THOR region, respectively. These variants were targeted in an allele-specific manner using sgRNA-guided dCas9-DNMT3A-3L complexes. In both cell lines, we successfully introduced DNA methylation specifically to the on-target allele of the *TERT* promoter with limited background methylation on the off-target allele or an off-target locus (*VEGFA*), respectively. We observed a maximum CpG methylation gain of 39% and 76% on the target allele when targeting the activating mutation and the common SNP, respectively. The epigenome editing translated into reduced *TERT* RNA expression in Hep-G2.

**Conclusions:**

We applied an ASEE-mediated approach to silence *TERT* allele specifically. Our results show that the concept of dominant oncogene inactivation by allele-specific epigenome editing can be successfully translated into cancer models. This new strategy may have important advantages in comparison with existing therapeutic approaches, e.g., targeting telomerase, especially with regard to reducing adverse side effects.

**Supplementary Information:**

The online version contains supplementary material available at 10.1186/s13148-023-01599-2.

## Background

The activation of dominant oncogenes by small variants (e.g., single-nucleotide variants or indels) or structural genomic alterations (e.g., copy number or structural variants) is a frequent driving mechanism common in many cancers [1, 2]. Moreover, some dominant oncogenes play a role in a broad range of different cancer types, e.g., common mutations affecting the *RAS* [3–5] signaling pathway or the *TERT* locus [6, 7]. Thus, silencing of dominant oncogenes, particularly those active in various cancer types, is an attractive strategy in cancer treatment. Ideally, such silencing should target only the dominantly activated oncogenic allele and leaves the wildtype allele intact in order to reduce potential side effects.

Epigenome editing by targeted DNA methylation alteration has been rapidly evolving over the last decade after the introduction of a clustered regularly interspaced short palindromic repeat (CRISPR)/dCas9 system, in which the Cas9 has been deprived of its ability to cleave DNA [8–11]. Recently, we have developed a super-specific way of ASEE [12]. Here, we set out to use this new approach in a proof-of-principle study to silence a dominant oncogene in an allele-specific manner. The specificity for the oncogenic allele in this technique is achieved by targeting a heterozygous sequence variant. On the one hand, this design allows directly targeting alleles carrying activating small variants. On the other hand, common heterozygous polymorphic single-nucleotide variants linked to an oncogenic allele make it possible to address also other genomic alterations like structural variants, e.g., oncogenic copy number gains or amplifications. We here chose the *TERT* locus encoding a subunit of telomerase for our proof-of-principle study.

Telomerase is an enzyme that adds telomeric repeats (TTAGGG) at the chromosomal ends, providing chromosomal stability to the cell during cell replication [13–15]. The two main components of telomerase holoenzyme are the catalytic subunit telomerase reverse transcriptase (TERT) and the telomerase RNA component (TERC) [16]. Telomerase activity is absent in most normal somatic cells but present in most human cancer cells facilitating cancer progression by telomere length maintenance [17, 18]. There are several mechanisms that lead to *TERT* activation that vary among the different types of cancers and include: chromosomal rearrangements involving the *TERT* gene [19–22], *TERT* transcriptional activation via transcription factor binding [23–25], miRNA regulation [26], DNA methylation changes at different elements of the *TERT* promoter [27, 28] and finally *TERT* promoter single nucleotide polymorphisms (SNPs) and recurrent mutations [6, 7, 29, 30]. The latter can lead to *TERT* expression by generating novel ETS transcription factor-family binding sites [6, 7, 29, 31]. Indeed, the *TERT* promoter region has been described as one of the “most frequently mutated non-coding cancer driver” [32, 33].

*TERT* promoter mutations are activating mutations which lead to *TERT* reactivation and expression from the mutated allele in cancer [34]. Moreover, the DNA methylation pattern of the *TERT* promoter region has been extensively studied in solid cancers and an association with gene expression has been shown [27, 35, 36]. Low DNA methylation of the *TERT* core promoter seems to be a prerequisite for *TERT* expression [36]. However, it has been demonstrated that high DNA methylation in a separate region further upstream is a hallmark of several cancer entities, including breast, brain and colon cancer, with the majority of malignancies in these entities being hypermethylated in this area [27, 36, 37]. Reporter assays in cervix and brain cancer cell lines revealed a significant drop in *TERT* expression, when this upstream region was unmethylated [27]. This led to the term *TERT* Hypermethylated Oncological Region (THOR) [27].

Using two different cell line models of common cancers, we here show that ASEE is suitable to efficiently and super-specifically modify DNA methylation at the *TERT* promoter region in an allele-specific manner using pathogenic sequence variants or polymorphic single-nucleotide variants as targets.

## Results

### In vitro* TERT* promoter screening in cell lines

**Fig. 1:**
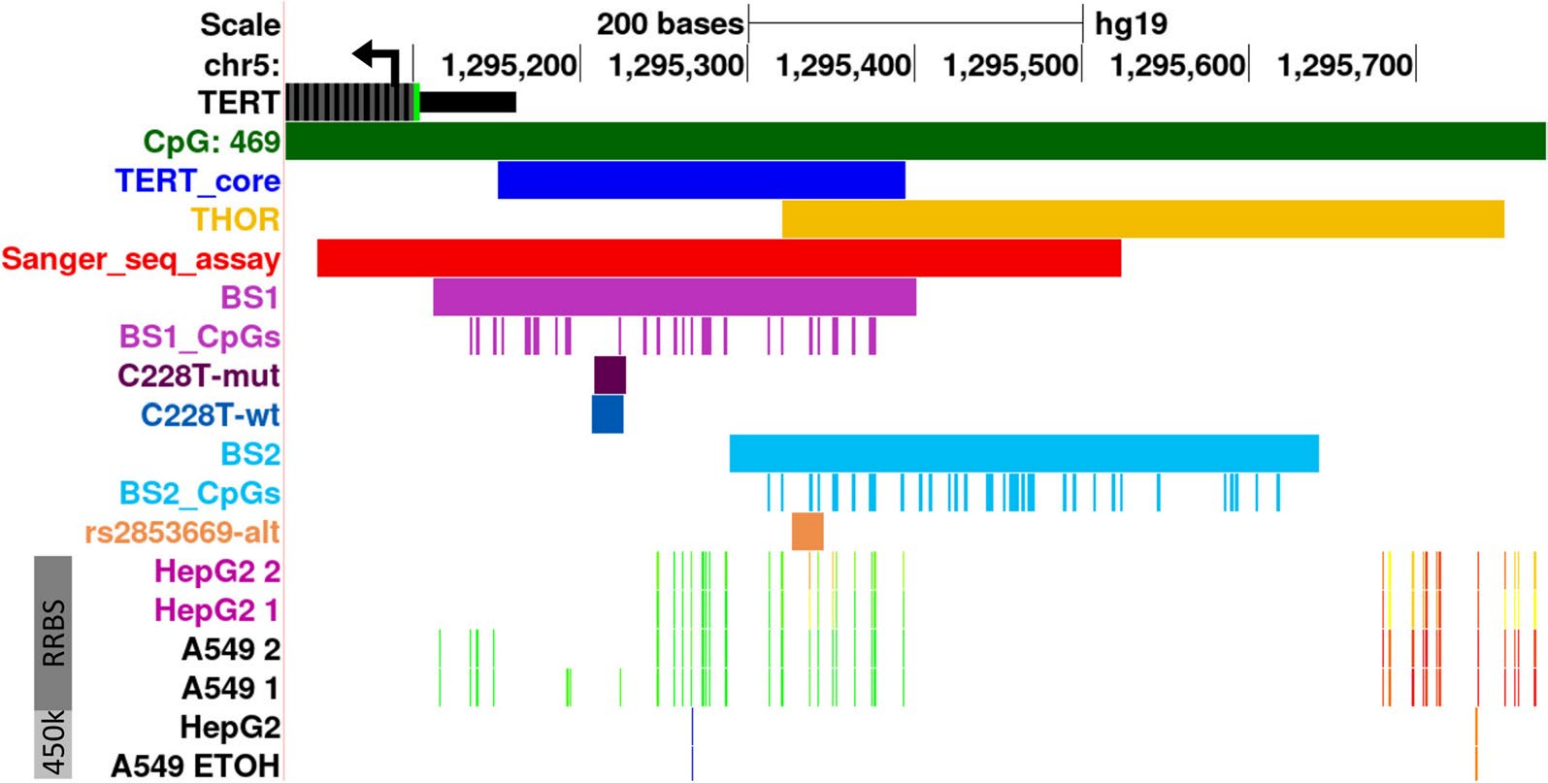
UCSC Browser view of *TERT* promoter region. The 5’ end of *TERT* gene is shown in black with arrow indicating the transcription direction. Dark green-colored track shows the CpG Islands track from UCSC browser. Blue-colored track depicts the *TERT* core promoter and yellow-colored track depicts the THOR region as described in Lee et al. 2019 [27]. Red-colored track shows the *TERT* promoter region which was screened in the cohort of 87 cell lines for SNVs by Sanger Sequencing. Light purple-colored tracks show the extend of the first *TERT* BS assay (BS1) used in Hep-G2 and A-549 and the 30 CpG sites included in the assay. Dark purple and dark blue tracks show the *TERT* sgRNAs [targeting both mutated (C228T-mut) and wildtype (C228T-wt) alleles of the C228T mutation] binding sites. Light blue-colored tracks show the extend of the second *TERT* BS assay used in A-549 cells (BS2) and the 35 CpG sites included in this assay. Orange track shows the binding site of the sgRNA that targets the alternative G allele of the rs2853669 common SNP (rs2853669-alt). At the lower part of the figure, Hep-G2 and A-549 DNA methylation tracks from the UCSC database are depicted. The DNA Methylation by Reduced Representation Bisulfite Sequencing (RRBS) from ENCODE/HudsonAlpha track is highlighted by a dark gray box on the left (DNA methylation status is represented with an 11-color gradient using the following scales: red = 100%, yellow = 50% and green = 0% of molecules sequenced are methylated). CpG Methylation by Infinium Human Methylation 450 K BeadChip arrays (450 k) from ENCODE/HAIB track is highlighted by a light gray box (orange = methylated [score ≥ 600] and blue = unmethylated [0 < score ≤ 200], where the score has a range of 0–1000)

A technical prerequisite for the application of ASEE is the presence of a heterozygous sequence variant which is needed as hook to direct the ASEE constructs to the target allele. To this end, we initialized the study by screening a genomic region containing the *TERT* core promoter and a part of THOR (Fig. 1) for sequence variants. From the 77 evaluable cell lines, 41 were heterozygous for at least one single-nucleotide variant (SNV), which renders 53% of the evaluable cell lines applicable for *TERT* ASEE (Additional file 1: Table S1). Most of the cell lines with at least one SNV were heterozygous for the common SNP rs2853669 (34/41). Interestingly, 7/34 of the cell lines showed a dominance of the alternative G allele at the rs2853669 position (Additional file 1: Table S1). Additionally, we detected the activating C228T (chr5:1,295,228, hg19) mutation in Hep-G2, which has already been described before [6, 7, 38].

For investigating the suitability of ASEE, we chose the Hep-G2 cell line due to its heterozygosity at the above described activating *TERT* promoter mutation. On the other hand, we employed the A-549 cell line which is heterozygous for the common SNP rs2853669 in the target region. Both described variants were chosen to serve as anchor for ASEE-induced silencing. In addition, HEK293 cells were used as control cells to set up the EpiEditing system.

### Establishment of epigenome editing at the *TERT* locus in HEK293 cells

We evaluated feasibility of targeted DNA methylation at the *TERT* locus with the designed constructs in HEK293, which does not carry the activating mutation C228T and is homozygous for the C allele. To this end, we performed transient transfection experiments with a three-plasmid system containing a catalytically deactivated Cas9 (dCas9) fused to a 10X SunTag peptide chain, a DNMT3A-3L R887E mutant with reduced DNA affinity [39] and a sgRNA that targets the C228T position (Additional file 1: Table S2). For this mutation position, we had two sgRNAs available: one sgRNA that targets the wildtype allele (hereafter termed as C228T-wt) and one sgRNA that targets the mutated allele (hereafter termed as C228T-mut). Since HEK293 is homozygous for the wildtype C allele, we employed the C228T-wt for the transfection experiments. The Cas9 enzyme recognizes a NGG PAM site during DNA binding [40]. Because the NGG site was located on the opposite strand of the C228T mutation, the sgRNAs were designed to target the G allele (which corresponds to C wildtype allele) and the A allele (which corresponds to T mutated allele).

Each of these vectors expressed a different fluorescent marker used for FACS. The dCas9 vector expressed TagBFP, the DNMT3A-3L vector expressed sfGFP, and the sgRNA vectors expressed DsRed fluorescent protein (Additional file 1: Table S2). The cells containing all three components of the system (triple-positive) were isolated and used for further analysis.

The triple transfection efficiency (i.e., transfection of all three plasmids) of HEK293 was 9.3% (± 3.5 standard deviation [SD]). Highest DNA methylation gain was observed at 50 bp up- and downstream of the C228T-wt sgRNA binding region (Additional file 1: Figure S1). In this area, we achieved an average DNA methylation gain of 19% after transfection with sgRNA C228T-wt when compared to the untreated samples and a maximum of 38% at CpG site 15 (position 156 in *TERT* bisulfite sequencing [BS] assay 1 [BS1], Fig. 1, Additional file 1: Figure S1A). The DNA methylation at the control *VEGFA* locus remained comparably low after targeted DNA methylation at the *TERT* locus. The average DNA methylation gain over the whole *VEGFA* assay was ~ 4% when compared to the untreated samples (Additional file 1: Figure S1B). To evaluate stability of epigenome editing, we analyzed DNA methylation at the *TERT* promoter 8 days post-transfection in HEK293 and observed the same levels as 3 days post-transfection (Additional file 1: Figure S1A) confirming long lasting effects.

These results show that we can deliver enduring targeted DNA methylation at the *TERT* core promoter in HEK293 cells with minor off-target locus effects.

### Introduction of allele-specific DNA methylation in Hep-G2 cells

After establishment of epigenome editing at the *TERT* locus in HEK293, we set out to utilize the three-plasmid system in another cell line system. For this purpose, we co-transfected Hep-G2 cells with the dCas9 and DNMT3A-3L R887E vectors mentioned above and one of the following sgRNA expression vectors: C228T-wt and C228T-mut (Additional file 1: Table S2). To control for unspecific DNA methylation, we performed transfection experiments with a scrambled sgRNA, which had minimum resemblance to the human genome [39]. We conducted at least three independent repeats for all experiments. Over all transfection experiments, we achieved an average triple transfection efficiency of 2.2% (± 1 SD) in Hep-G2.

**Fig. 2:**
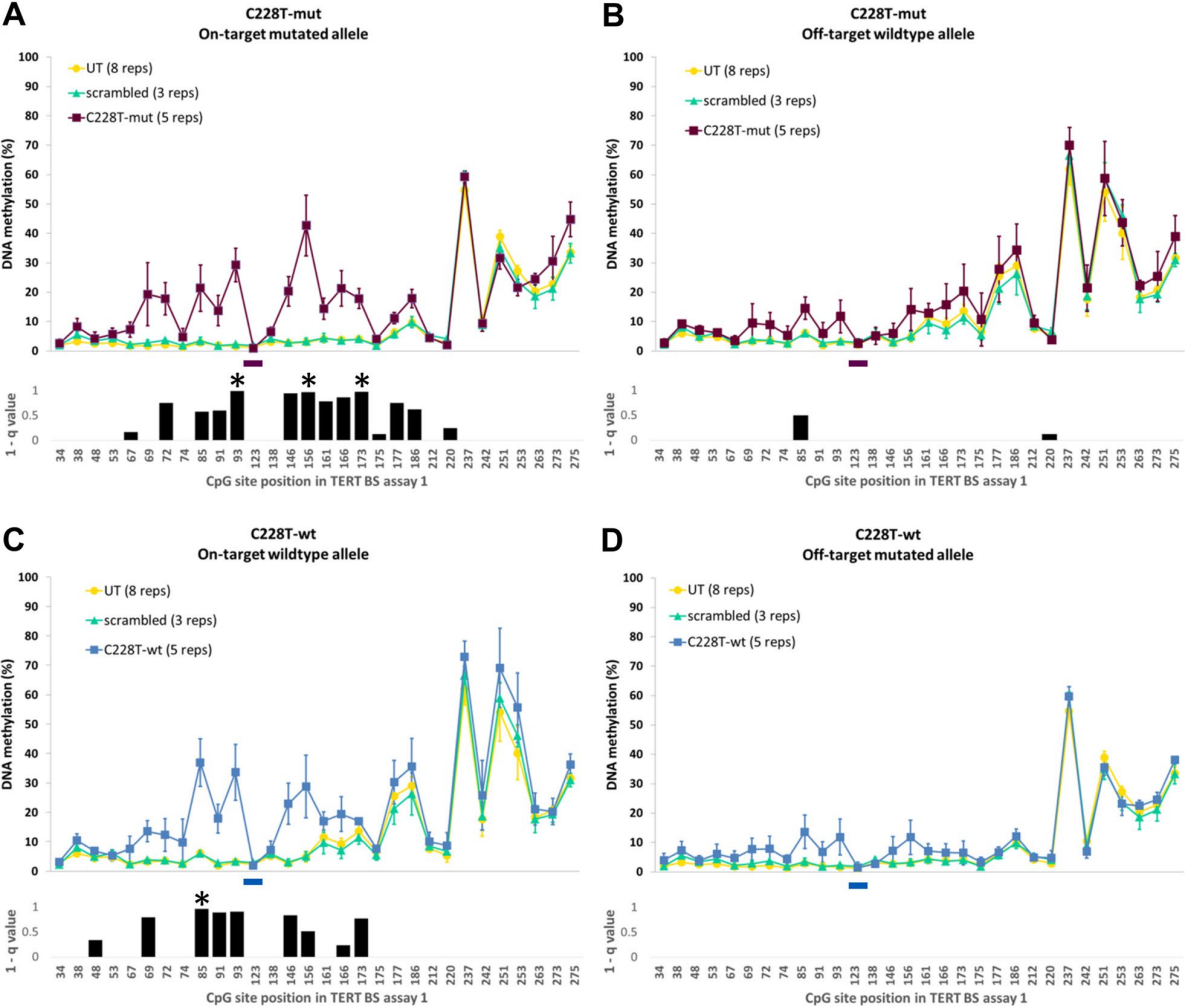
*TERT* promoter DNA methylation in Hep-G2 after ASEE targeting the C228T mutated and wildtype allele. Average allele DNA methylation after ASEE with sgRNAs targeting the C228T position in Hep-G2 (**A**) sgRNA C228T-mut, on-target mutated allele is shown in purple; (**B**) off-target allele shown in purple; (**C**) sgRNA C228T-wt, on-target wildtype allele shown in blue; (**D**) off-target mutated allele shown in blue. Untreated Hep-G2 cells (UT) are shown in yellow and Hep-G2 transfected with scrambled sgRNA are shown in light green. The x-axis shows the position of CpG sites in the BS1 assay according to the reference sequence used in the analysis. **A**, **B** The purple line at position 123 shows the binding site for sgRNA C228T-mut. **C**, **D** The blue line at position 123 shows the binding site for sgRNA C228T-wt. Error bars are calculated based on at least three independent experiments. Black bar plots show the 1-q value after T Test comparison between Hep-G2 transfected with C228T-mut (A, B) or C228T-wt (**C**, **D**) and Hep-G2 transfected with scrambled sgRNA. q values > 1 were set as 1 for visualization purposes. Highly significant CpG sites are indicated by stars (q < 0.05)

Similar to the HEK293 experiments, we observed the highest on-target allele DNA methylation gain at 50 bp up- and downstream of the sgRNA binding sites (Fig. 2A, C). In this area, we introduced after transfection with sgRNA C228T-mut an average DNA methylation gain of 16% with a maximum of 39% at CpG site 15 on the on-target allele (position 156 in BS1, Fig. 2A). This result is well above the average DNA methylation gain of 5% on the respective off-target allele (Fig. 2B). When targeting the wildtype allele with sgRNA C228T-wt, we introduced a mean DNA methylation gain of 15% at 50 bp up- and downstream of the sgRNA binding site with a maximum of 31% at CpG site 9 (position 85 in BS1, Fig. 2C) compared to an average DNA methylation gain of 5% on the respective off-target allele (Fig. 2D). Hep-G2 transfected with scrambled sgRNA showed no changes in DNA methylation levels compared to untreated Hep-G2 cells (Fig. 2). Additionally, we examined the DNA methylation in 12 CpGs at the *VEGFA* promoter region which served as an off-target DNA methylation control locus. This analysis showed an average overall DNA methylation gain of ~ 2% for C228T-mut and ~ 1% for C228T-wt as compared to the untreated Hep-G2 and less than 1% in comparison with Hep-G2 transfected with scrambled sgRNA (Additional file 1: Figure S2A). However, we observed an average DNA methylation gain of 7% at a single CpG site within the *VEGFA* locus in all the treated samples when compared to the untreated Hep-G2 (CpG 177, Additional file 1: Figure S2A).

Together the results show that our ASEE complexes can indeed deliver DNA methylation specifically to both individual alleles of the target region (*TERT* core promoter) with limited effects on the off-target allele and off-target locus.

### ASEE in A-549 shows allele-specific DNA methylation when targeting the alternative G allele at the rs2853669 SNP position

**Fig. 3:**
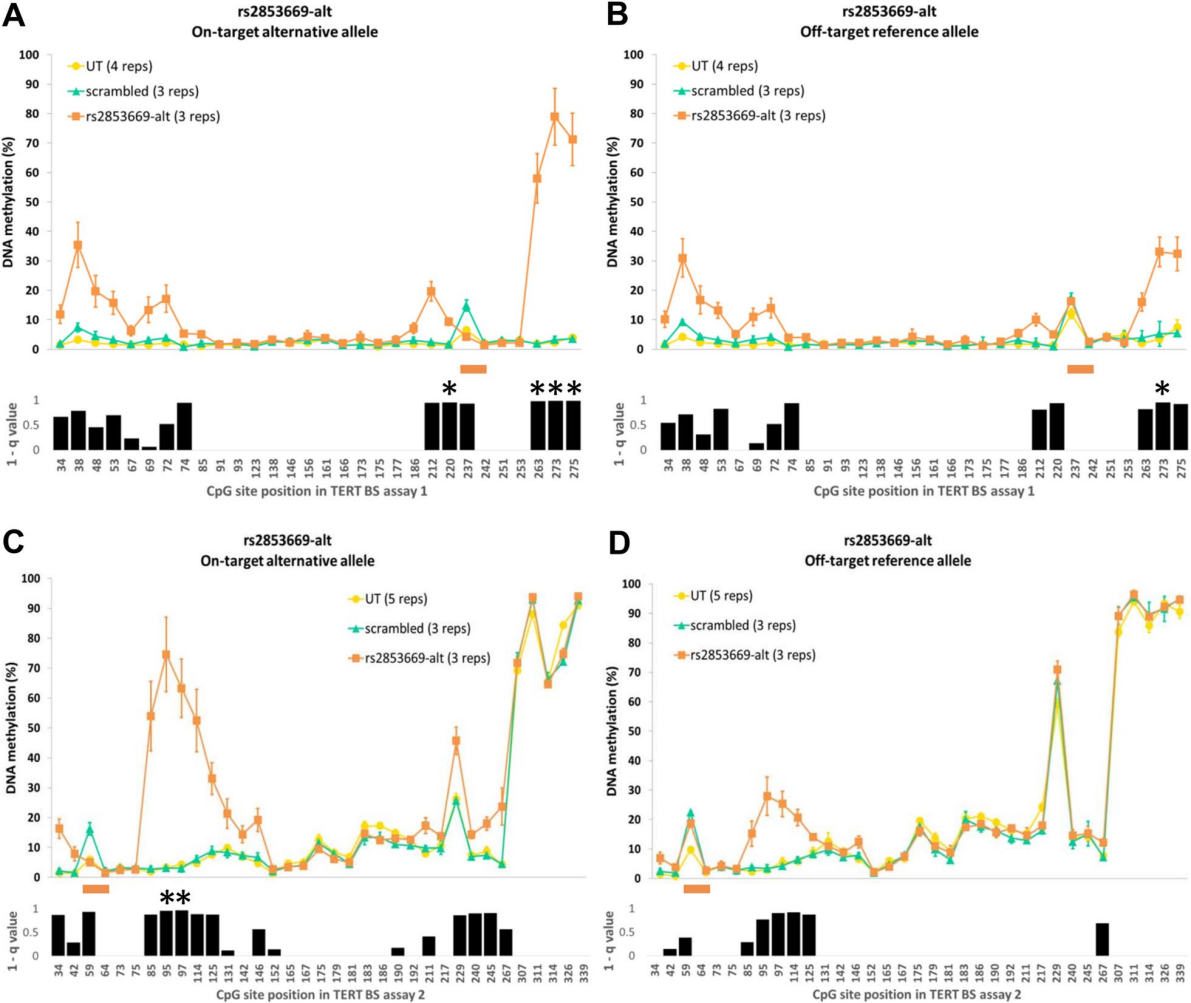
*TERT* promoter DNA methylation in A-549 after ASEE targeting the alternative allele at rs2853669 SNP. Average allele DNA methylation in A-549 after ASEE with sgRNA targeting the G alternative allele at rs2853669 SNP position. **A**, **C** sgRNA rs2853669-alt, on-target alternative G allele orange; **B**, **D** off-target reference A allele orange. **A**, **B** The x-axis shows the position of CpG sites in the BS1 assay according to the reference sequence used in the analysis and the orange line at positions 237–242 shows the binding site for sgRNA rs2853669-alt. **C**, **D** The x-axis shows the position of CpG sites in the BS2 assay according to the reference sequence used in the analysis and the orange line at positions 59–64 shows the binding site for sgRNA rs2853669-alt. **A**–**D** Untreated A-549 (UT) are shown in yellow and A-549 transfected with scrambled sgRNA are shown in light green. Error bars are calculated based on at least three independent experiments. Black bar plots show the 1-q value after T Test comparison between A-549 transfected with rs2853669-alt and A-549 transfected with scrambled sgRNA. q values > 1 were set as 1 for visualization purposes. Highly significant CpG sites are indicated by stars (q < 0.05)

We next set out to evaluate ASEE in a different cancer cell line now addressing the THOR region of the *TERT* promoter using a frequent common SNP for allele discrimination (Fig. 1). We co-transfected A-549 cells with a mix of the three plasmids that contained vectors expressing dCas-10X SunTag system, DNMT3A-3L R887E mutant and a sgRNA that targets the alternative allele G at the rs2853669 SNP, hereafter termed rs2853669-alt (Additional file 1: Table S2). We observed an average triple transfection efficiency of 1.7% (± 0.5 SD) in this cell line. First, we analyzed the BS1 assay in A-549 (Fig. 3A, B). We observed the highest on-target allele DNA methylation effect mainly 35 bp up- and downstream of the sgRNA binding site and this effect was highly significant with q values < 0.05 (Fig. 3A). In this area, we delivered an average DNA methylation gain of 32% after transfection with sgRNA rs2853669-alt with a maximum gain of 76% at CpG site 29 (position 273 in BS1, Fig. 3A) as compared to an average DNA methylation gain of 11% on the off-target allele (Fig. 3B). As compared to transfection with sgRNA C228T-mut in Hep-G2, off-target wt allele DNA methylation gain was higher in A-549 (5% versus 11%). This could be due to higher residual binding of the sgRNA rs2853669-alt/dCas9 complex at the off-target allele as compared to the sgRNA C228T-mut/dCas9 complex, e.g., due to different chromatin accessibility at the respective regions. A-549 transfected with scrambled sgRNA did not show changes in DNA methylation levels compared to untreated A-549 cells (Fig. 3). Because the DNA methylation effect seemed to spread further upstream of the region covered with BS1, we designed an overlapping second assay (BS2) to examine the adjacent upstream DNA region (Fig. 1). Indeed, we observed a spread of the ASEE effect covering 60 bp upstream the sgRNA binding site (Fig. 3C, D). Nevertheless, the maximum gain of DNA methylation was already observed in the BS1 assay. In line with the published high DNA methylation levels in the THOR in A-549, we detected high DNA methylation levels toward the end of BS2 that covers this region (compare Fig. 1). Again, we examined the same 12 CpG sites at the *VEGFA* promoter region mentioned above. This analysis showed an average overall DNA methylation gain of ~ 2% for rs2853669-alt sgRNA as compared to the untreated A-549. When we compared against the A-549 transfected with scrambled sgRNA, these values were less than 0.5% (Additional file 1: Figure S2B). As also shown in Hep-G2, we observed the same DNA methylation increase at the same single CpG site 177 with an average gain of 11% in all treated samples when compared to the untreated A-549 cells (Additional file 1: Figure S2B). Thus, we showed that our ASEE system was successfully repurposed to target a common SNP at the TERT promoter in a different cell line with negligible effects on the off-target allele and off-target locus.

### Effects of ASEE of *TERT* promoter on *TERT* RNA expression

**Fig. 4:**
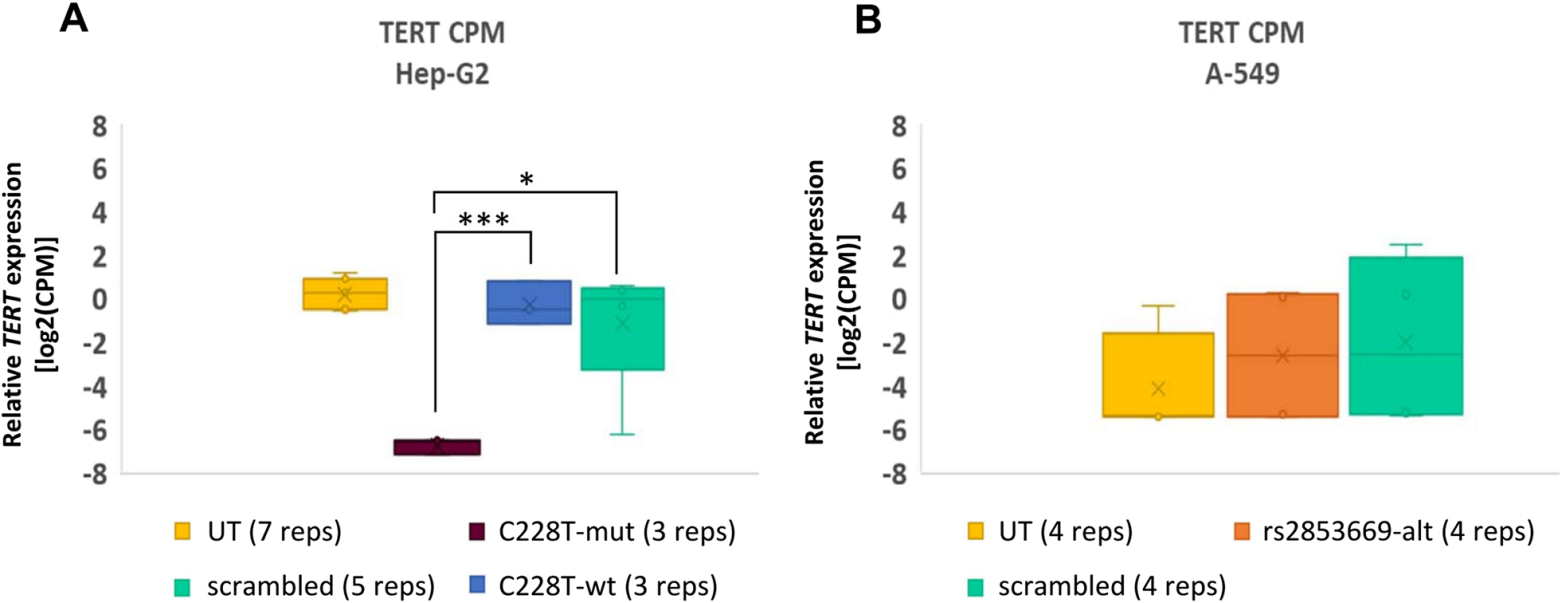
Box plots showing *TERT* RNA expression after ASEE in Hep-G2 and A-549. **A** *TERT* RNA expression shown as log2[CPM] values in untreated Hep-G2 and Hep-G2 transfected with different sgRNAs. Purple and blue color corresponds to *TERT* sgRNA targeting the mutated (C228T-mut) and wildtype (C228T-wt) allele at C228T position respectively. **B** *TERT* RNA expression shown as log2[CPM] values in untreated A-549 and A-549 transfected with different sgRNAs. Orange color corresponds to *TERT* sgRNA targeting the alternative G allele at rs2853669 SNP position (rs2853669-alt). **A**, **B** Yellow color corresponds to untreated cells (UT) and light green color corresponds to cells transfected with scrambled sgRNA

To analyze the effect of ASEE, we determined RNA expression via the HTP panel assay. In Hep-G2 cells, *TERT* showed a highly significant 20-fold decrease of RNA expression in the samples which were transfected with C228T-mut as compared to C228T-wt (p value < 0.0005) and sixfold decrease as compared to scrambled sgRNA (p value < 0.05) (Fig. 4A). *TERT* expression levels were not significantly different between untreated Hep-G2 and Hep-G2 transfected with C228T-wt (Fig. 4A). *VEGFA* expression levels in Hep-G2 were similar among untreated and all transfected samples (Additional file 1: Figure S2C). Regarding A-549 cells, there was no significant change on *TERT* RNA expression after successful ASEE at the common SNP rs2853669 (Fig. 4B). *VEGFA* RNA expression in A-549 remained also unchanged after ASEE and transfection with scrambled sgRNA (Additional file 1: Figure S2D). These results prove the concept that DNA methylation delivery to the *TERT* allele harboring the activating promoter mutation in the core promoter is indeed associated with reduced *TERT* mRNA expression.

### ASEE effect on cell viability in Hep-G2 and A-549 cells

**Fig. 5:**
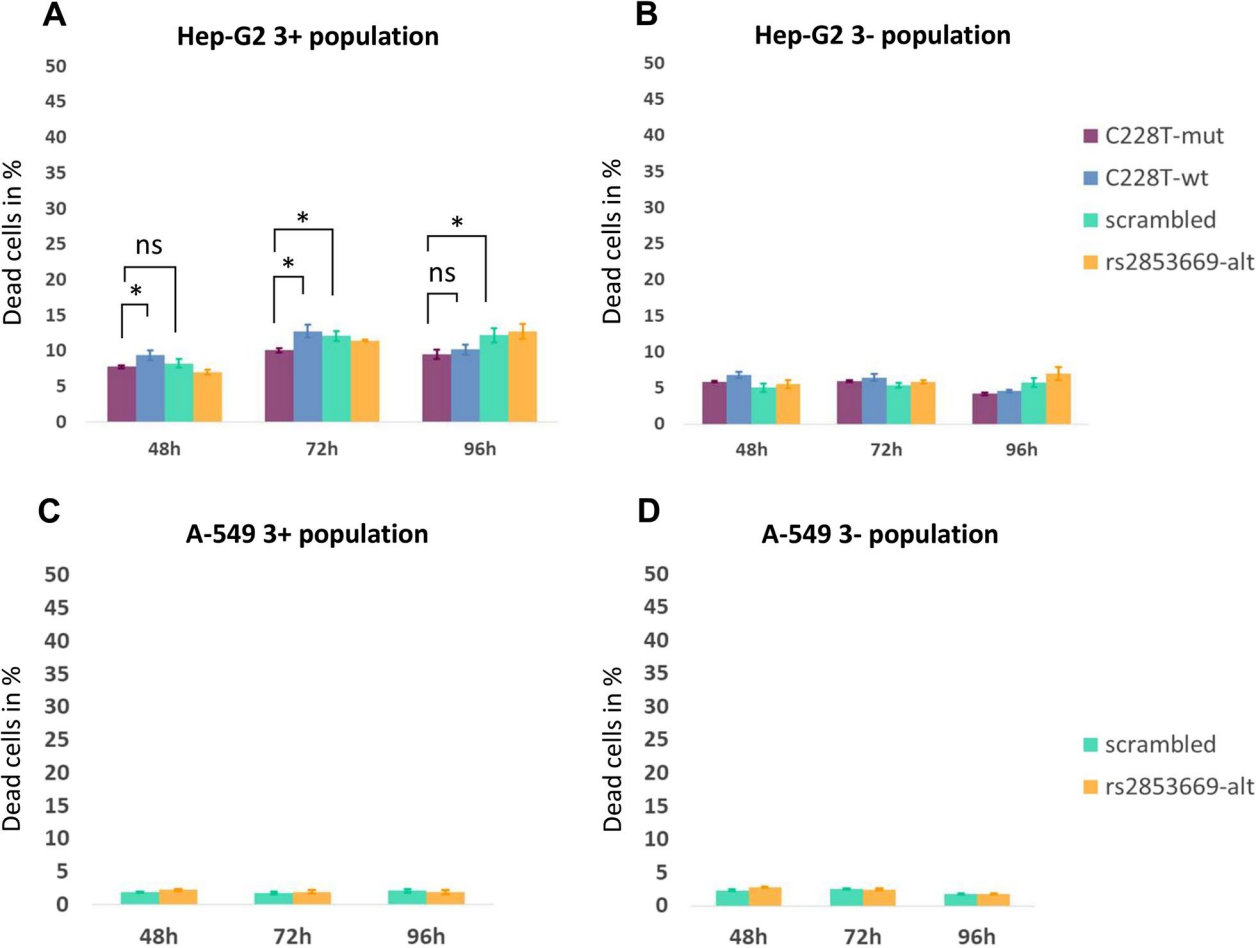
Viability of Hep-G2 and A-549 after ASEE, analyzed by flow cytometry with Zombie NIR system. Bar plot showing the percentage (%) of dead cells within the Hep-G2 cell population (**A**) and A-549 cell population (**C**) carrying all three components (triple-positive for dCas9-10X Suntag vector, DNMT3A-3L vector and sgRNA vector). Bar plot showing the percentage (%) of dead cells within the Hep-G2 cell population **B** and A-549 cell population **D** carrying none of the three components (triple negative). **A**–**D** Purple and blue color corresponds to cells transfected with *TERT* sgRNA targeting the mutated (C228T-mut) and wildtype (C228T-wt) allele at C228T position respectively, light green color corresponds to cells transfected with scrambled sgRNA, and orange color corresponds to cells transfected with *TERT* sgRNA targeting the G alternative allele at rs2853669 SNP position (rs2853669-alt)

Next, we explored cell viability after ASEE establishment. Over all transfection experiments in Hep-G2, we observe a higher rate of dead cells among the triple-positive cells containing all three plasmids (7–13%) compared to the untreated cells (6%) (p < 0.001, Fig. 5A, B). The same death rate levels were observed in the single-positive populations (7–14%, Additional file 1: Figure S3A). Thus, transfection itself has a negative effect on cell survival. For the triple-negative cells, the death rate was the same among all *TERT* sgRNA and scrambled sgRNA experiments (4–7%, Fig. 5B). Flow cytometry analysis on A-549 cells showed no difference in the rate of dead cells between the triple-positive and the triple-negative populations (p > 0.05) and between the single-positive populations within the same sample (Fig. 5C, D, Additional file 1: Figure S3B). Thus, we did not observe a specific effect of epigenome editing on cell survival in the time window available for analysis.

## Discussion

In the present study, we successfully introduced DNA methylation specifically to one allele of the *TERT* promoter in two cancer cell lines with limited effects at the second allele. Nunez and colleagues systematically performed an evaluation of the capacities of a CRISPR-based programmable epigenome editor protein, called CRISPRoff and CRISPRon, that allowed heritable depletion and introduction of DNA methylation to control gene expression (41). They showed highly specific and robust gene silencing with CRISPRoff, consisting of a single dead Cas9 fusion protein dCas9-DNMT3A-3L, with the advantage of rapid reversion of DNA methylation if desired [41]. Recently, a related technique has been further developed into a super-specific highly efficient allele-specific epigenome editing complex [12]. The present study takes advantage of this new development and successfully translates the idea to silence dominantly activated oncogenic alleles selectively by ASEE into relevant cancer models.

We observed different downstream effects of DNA methylation gain in the *TERT* promoter in the two cell lines examined. Several variables might have an influence on this observation. Firstly, we addressed different sequence variants. Hep-G2 harbors an activating *TERT* promoter mutation that leads to monoallelic overexpression equaling biallelic expression in other cancer entities such as melanoma [34]. Thus, we targeted the source of *TERT* expression in Hep-G2. In A-549 *TERT*, expression does not rely on the targeted *TERT* promoter variant itself but rather is driven by the CTCF transcription factor, which interacts with a distal *TERT* enhancer element [31, 42]. It has been shown previously that direct RNA targeting approaches (RNAi) in A-549 cells led to reduced telomerase activity and apoptosis in vitro and reduced tumorigenic potential in vivo [43]. While this is an alternative approach, it does not allow allele-specific alterations. Nevertheless, these results support A-549 to be a promising cancer model for *TERT* silencing. Our approach does not necessarily need to target directly the source of the oncogene activation. It can take advantage of a tag SNP to silence gene expression of the oncogenic allele. Of interest, the common SNP rs2853669 that served for allele discrimination in A-549 has been associated with increased lung cancer risk in Asian populations [44]. However, this SNP was also coupled with reduced *TERT* expression and better survival in the context of activating *TERT* promoter mutations in other cancer types such as glioblastoma and bladder cancer [45, 46]. Interestingly, rs2853669 destroys an existing ETS-factor binding site [30, 47]. Thus, this SNP likely plays a role in those tumors that activate *TERT* via transcriptional activators.

Next, due to the genomic location of the sequence variants used for allelic discrimination, we addressed different areas of the *TERT* promoter. In Hep-G2 cells, we targeted the core promoter. Introduction of DNA methylation to the core promoter of the mutated *TERT* promoter allele successfully inhibited *TERT* expression in Hep-G2. This is in line with the observation of Renaud et al., who report that a partial hypomethylation of the core promoter is necessary for *TERT* expression (48).

In A-549 cells, we targeted the THOR. In some solid cancers like HCC and brain cancer, high DNA methylation of THOR has been associated with *TERT* expression (49, 50). We did not observe TERT expression change upon editing of THOR in A-549. According to recent studies, hypomethylated THOR is an inhibitory element of the *TERT* promoter, as shown by luciferase reporter assays [37, 51]. Although a decrease of DNA methylation was observed using a dCas9-TET1 demethylation system on the MCF-7 breast cancer cell line, there was no significant effect on *TERT* RNA expression (51). In-depth investigations of the functional role of the different DNA methylation patterns in THOR in the cell lines used, was not the primary focus of the present study. Nevertheless, it could be interesting to explore the effects of TET-induced ASEE-mediated demethylation of the THOR on TERT expression in the cell lines analyzed herein.

Our main interest by designing this study was exploration of the potential of ASEE-mediated allele-specific *TERT* inhibition as proof of principle for silencing of other dominantly activated oncogenic alleles. While we show efficient and specific DNA methylation introduction into the *TERT* promoter with successful repression of *TERT* RNA transcription in Hep-G2, we did not observe an adverse effect on cell survival thereafter. This likely is due to the observation time window that did not exceed 96 h. Existing therapy with telomerase inhibitors like Imetelstat (direct oligonucleotide telomerase inhibitor) rely on cumulative telomere shortening before anticancer effects are exerted [52, 53]. Thus, a prolonged lag period is anticipated until ASEE could have an effect. Clinical trials on patients with solid tumors uncovered dose-limiting toxicity due to hematological side effects (thrombocytopenia, neutropenia) [54]. This might be due to the fact that telomerase was inhibited not only in cancer cells but also in stem and precursor cells of the hematopoietic lineages [54]. When targeting a somatic cancer mutation in the *TERT* promoter, the approach presented here could provide the key advantage of reducing adverse side effects of anti-TERT treatments, since the TERT inhibition is specific for the cancer cells harboring the SNV target for the ASEE complex. Thus, we think it is worthwhile to work on a translation of these results into clinical settings. Introduction of the ASEE complex into the target organs and sufficient transfection efficiency rates are among the main challenges that needs to be tackled. Moreover, the method needs further validation by whole genome bisulfite sequencing to analyze potential methylation changes genome-wide. However, we are confident that our approach provides promising results to work on overcoming these natural obstacles.

## Conclusions

We successfully applied here a super-specific ASEE approach and inhibited *TERT* mRNA expression by introducing an efficient DNA methylation gain to the *TERT* promoter allele carrying an activating *TERT* mutation. This strategy may have important advantages in comparison with existing telomerase-directed approaches with regard to reducing adverse side effects because the second allele of the target gene is not affected. On a more general level, this is to the best of our knowledge the first proof-of-principle study of a promising method to silence dominantly activated oncogenic alleles specifically by DNA methylation. This strategy may allow either targeting directly a pathogenic single-nucleotide variant or, e.g., in case of a copy number or structural variant, targeting a polymorphic single-nucleotide variant linked to the oncogenic allele.

## Methods

### Screening of cancer cell lines for SNVs as prerequisite for ASEE by Sanger sequencing

A 482 bp long region in the *TERT* promoter region (chr5:1,295,043–1,295,524, hg19) was investigated in order to detect heterozygous SNVs (activating mutations or SNPs) that could serve as a target for ASEE. To this end, DNA from 87 cell lines was extracted using the FlexiGene DNA Kit (QIAGEN, Venlo, Netherlands). Cell lines were selected according to availability and chances for successful transfection. Most of the cell lines screened in this study were lymphomas of B-cell origin in addition to adherent cell lines (hepatocellular carcinoma and lung adenocarcinoma). All DNA samples used in this study were authenticated using the GenePrint 10 System (Promega, Madison, Wisconsin, USA) according to the manufacturer’s instructions and cell lines were tested for mycoplasma contamination using the MycoSPY-RCR Mycoplasma Test Kit (Biontex, München, Germany) following the manufacturer’s protocol. Cell lines that were transduced or transfected were excluded from the screening. A total of 50 ng genomic DNA were used to amplify the *TERT* promoter region with the AmpliTaq Gold® 360 PCR Master Mix (Thermo Fisher Scientific, Waltham, Massachusetts, USA) and primers that contained universal tags for sequencing (Additional file 1: Table S3). The PCR was performed in a Labcycler Basic 011–103 (Sensoquest, Göttingen, Germany) and the conditions applied for TERT promoter region amplification were as follows: 10 min at 95 °C, 40 cycles of 5 s at 95 °C, 30 s at 61 °C, 30 s at 72 °C and finally 5 min at 72 °C. The PCR products were purified using the AMPure XP magnetic beads (Beckman Coulter Life Sciences, Brea, California, USA) following the manufacturer’s instructions. The purified PCR products subsequently underwent a sequencing reaction using the BigDye™ Terminator version 3.1 Cycle Sequencing Kit (Applied Biosystems™, Waltham, Massachusetts, USA) according to the manufacturer’s instructions. The sequencing reactions were purified with Agencourt CleanSEQ beads (Beckman Coulter Life Sciences, Brea, California, USA) according to the manufacturer’s protocol. Sequencing was performed on a 3500xL Dx Genetic Analyzer (Applied Biosystems, Waltham, Massachusetts, USA). The peak calling was done with the Sequencing Analysis Software version 5.4 (Applied Biosystems, Waltham, Massachusetts, USA) and finally the data were visualized with the Sequence Scanner Software 2 version 2.0 (Applied Biosystems, Waltham, Massachusetts, USA). From the 87 cell lines, 77 were evaluable for both strands. Only the sequence variants that were present in both DNA strands were considered.

### Plasmid propagation and validation by Sanger sequencing

In order to generate sufficient quantity of vectors for the transfection experiments, 5-alpha Competent E. coli bacteria (NEB, Ipswich, Massachusetts, USA) were transformed following the manufacturer’s guidelines. The dCas9 and DNMT expression vectors used in this study have been described before [39]. For positive clone selection after transformation, the bacteria grew in Luria broth base (LB), Miller′s modified medium (Sigma-Aldrich, St. Louis, Missouri, USA) supplemented with 100 mg/ml Ampicillin or 50 mg/ml Kanamycin (AppliChem, Darmstadt, Germany) according to the resistance cassette of each vector (Additional file 1: Table S2). Plasmid DNA was isolated using the NucleoBond Xtra Midi kit (Macherey Nagel, Düren, Germany) according to manufacturer’s instructions and measured with the Qubit dsDNA BR-Assay-Kit (Invitrogen, Waltham, Massachusetts, USA).

For plasmid validation, 50 ng of extracted plasmid was used to amplify unique parts of each plasmid with the AmpliTaq Gold® 360 PCR Master Mix (Thermo Fisher Scientific, Waltham, Massachusetts, USA) and primers that were specific for each vector (Additional file 1: Table S3). The PCR was performed in a Labcycler Basic 011–103 (Sensoquest, Göttingen, Germany) and the conditions applied were as follows: 15 min at 98 °C, 40 cycles of 30 s at 95 °C, 30 s at 61 °C, 30 s at 72 °C and finally 10 min at 72 °C. The PCR products were purified using the AMPure XP magnetic beads (Beckman Coulter Life Sciences, Brea, California, USA) following the manufacturer’s instructions and a sequencing reaction was performed as described above. Sequencing reactions were purified with Agencourt CleanSEQ beads (Beckman Coulter Life Sciences, Brea, California, USA) according to the manufacturer’s protocol and eventually sequenced using the 3500xL Dx Genetic Analyzer (Applied Biosystems, Waltham, Massachusetts, USA). The peak calling was done with the help of the Sequencing Analysis Software version 5.4 (Applied Biosystems, Waltham, Massachusetts, USA) and finally the data were visualized with the Sequence Scanner Software 2 version 2.0 (Applied Biosystems, Waltham, Massachusetts, USA).

### Cell culture

Hep-G2 hepatocellular carcinoma cells and HEK293 cells were cultivated in RPMI 1640 Medium 1X (GIBCO Life Technologies, Carlsbad, California, USA) supplemented with 10% Fetal Bovine Serum (FBS, GIBCO Life Technologies, Carlsbad, California, USA) and 1% L-Analyl-L-Glutamine (Biochrom, Merck Millipore, Burlington, Massachusetts, USA). A-549 lung carcinoma cell line was cultivated in DMEM high glucose medium (GIBCO Life Technologies, Carlsbad, California, USA) supplemented with 10% FBS (GIBCO Life Technologies, Carlsbad, California, USA). Every 3–4 days we detached the cells from the flask bottom using diluted Trypsin 2,5% w/v in PBS w/o Ca_2_ + (Biochrom, Merck Millipore, Burlington, Massachusetts, USA). All cells were incubated at 37 °C and 5% CO_2_ in a Heracell™ 240i CO_2_ Incubator (Thermo Fisher Scientific, Waltham, Massachusetts, USA).

### Co-transfection experiments and fluorescence activated cell sorting (FACS)

Transient transfection experiments were performed on Hep-G2 cells 24 h after seeding 850,000 cells/well in a 6-well plate (Thermo Fisher Scientific, Waltham, Massachusetts, USA). The Lipofectamine 3000 reagent (Thermo Fisher Scientific, Waltham, Massachusetts, USA) was utilized following the manufacturer’s instructions. The following vectors, each expressing a different fluorescent marker, were used for the co-transfection of Hep-G2: dCas9-10X SunTag system (10,032 bp; TagBFP expressing), a DNMT3A-3L R887E mutant with reduced DNA affinity (6,200 bp; sfGFP expressing) [39] and a sgRNA that targets either the wildtype or the mutated allele at the C228T position (5,097 bp; DsRed expressing), hereafter termed C228T-wt and C228T-mut respectively (Additional file 1: Table S2). Because the NGG site was located on the opposite strand of the C228T mutation, the sgRNAs were designed to target the G allele (which corresponds to C wildtype allele) and the A allele (which corresponds to T mutated allele). Development, optimization and validation of these constructs are described elsewhere [12]. Upon expression of the dCas9-10X SunTag fused protein, this complex can recruit up to 10 active subunits of DNMT3A-3L [39]. All vectors were validated by Sanger sequencing prior to the transfection experiments. For the A-549 cell line transfection, 500,000 cells/well were seeded in four 6-well plates (Thermo Fisher Scientific, Waltham, Massachusetts, USA) 24 h prior to the transfection experiment. The same vectors as in the Hep-G2 experiments were employed with a different sgRNA that targets the alternative G allele of the SNP rs2853669, hereafter termed rs2853669-alt. Additionally, the non-cancer cell line HEK293 was used to establish transfection experiments. A total of 250,000 HEK293 cells/well were seeded in three wells (three technical replicates) of a 6-well plate (Thermo Fisher Scientific, Waltham, Massachusetts, USA) 24 h prior to the transfection experiment. The FuGENE HD transfection reagent was used for HEK293 experiments according to the manufacturer’s instructions (Promega, Madison, Wisconsin, USA). Since this cell line is homozygous for the wildtype C allele at C228T position, C228T-wt was used, thereby addressing both alleles. The cells were harvested by trypsinization 72 h post-transfection and filtered through a 35 µm cell strainer cap (FALCON, Corning, New York, USA). Cells that contained all three components (triple-positive) were isolated by FACS with a BD FACSAria™ III Cell Sorter (BD Biosciences, New Jersey, USA). The ranges of triple-positive cells sorted were: 45,000–295,000 for HEK293, 69,000–400,000 for Hep-G2 and 60,000–147,000 for A-549. These cells were later on handled for downstream analysis including DNA isolation, bisulfite conversion, library generation and sequencing.

### Bisulfite treatment and targeted bisulfite sequencing (BS)

After isolation of triple-positive Hep-G2, A-549 and HEK293 cells, genomic DNA was extracted using the Quick-DNA/RNA™ Microprep Plus Kit (Zymo Research, Irvine, California, USA) following the instructions of the manual. A total of 1,000 ng of genomic DNA was used for bisulfite conversion and purification with the EpiTect Bisulfite Kit (QIAGEN, Venlo, Netherlands) according to the manufacturer’s instructions. The purified bisulfite converted DNA was eluted in a final volume of 20 μL. The following regions of the *TERT* promoter were screened: BS1 (chr5:1,295,112–1,295,401, hg19, 290 bp, 30 CpGs) used in all three cell lines and BS2 (chr5:1,295,290–1,295,642, hg19, 353 bp, 35 CpGs) used only in A-549 cells. The last nine CpGs of the BS1 assay overlapped with the first nine CpGs of the BS2 assay (Fig. 1). For the PCR amplification of the *TERT* promoter region, 1 μL of bisulfite converted DNA was set up, 12.5 μL of the PyroMark PCR mix from the PyroMark PCR Kit (QIAGEN, Venlo, Netherlands) and 10 pmol of each primer containing the overhang adapters from the 16S Metagenomic Sequencing Library Preparation protocol (Illumina, San Diego, California, USA). The PCR was conducted in a Labcycler Basic 011–103 (Sensoquest, Göttingen, Germany) with the following conditions for *TERT* BS1: 15 min at 98 °C, 7 cycles of 30 s at 98 °C, 30 s at 58–55 °C (dT -0,5/cycle), 30 s at 72 °C, 38 cycles of 30 s at 98 °C, 30 s at 55 °C, 45 s at 72 °C and finally 10 min at 72 °C. For *TERT* BS2 assay the following PCR conditions were used: 15 min at 95 °C, 35 cycles of 30 s at 94 °C, 30 s at 55 °C, 30 s at 72 °C and finally 10 min at 72 °C.

The *VEGFA* promoter locus (chr6:43,738,171–43,738,372, hg19, 202 bp, 12 CpGs) was selected as an off-target DNA methylation control since it has been shown that it is a sensitive region whose CpG island is easily methylated by epigenome editing systems [39]. The PCR conditions applied for *VEGFA* promoter amplification were as follows: 15 min at 95 °C, 45 cycles of 30 s at 94 °C, 30 s at 50 °C, 30 s at 72 °C and finally 10 min at 72 °C. The PCR products were purified with AMPure XP magnetic beads (Beckman Coulter Life Sciences, Brea, California, USA). Indexed PCR products were generated using the IDT for Illumina UD Indexes Plate Set A (Illumina, San Diego, California, USA) and the EPM Enhanced PCR Mix (Illumina, San Diego, California, USA) in a Biometra thermocycler (Jena Analytik, Jena, Germany) with the following PCR conditions: 3 min at 72 °C, 3 min at 98 °C, 9 cycles of 20 s at 98 °C, 30 s at 60 °C, 1 min at 72 °C and finally 3 min at 72 °C. Thereafter, the indexed PCR products were purified with the same magnetic beads mentioned above and 100 ng of each purified library were used to create pools for NGS. Targeted bisulfite sequencing was performed on an Illumina MiSeq sequencer (Illumina, San Diego, California, USA) using a paired-end 2 × 300 cycles protocol. The bisulfite conversion rate was calculated based on the ratio of total unmethylated C’s outside of CpG context to the sum of total methylated and unmethylated C’s outside of CpG context.

To control for amplification bias of one allele, unique molecular identifiers (UMIs) were added to the forward primer sequence. These UMIs allow quantification of the original DNA fragments among the final sequencing reads (Additional file 1: Table S3). The length of each UMI is six nucleotides, resulting in a maximum of 4,096 unique UMI per sequencing experiment. Therefore, it is expected that several UMIs are found more than once if thousands (i.e., > 4,096) of reads per allele were sequenced. In Hep-G2 and A-549 cell lines, we observed all possible UMI sequences (minimum complexity of 4,096 molecules) and we did not observe higher frequency than 50 reads per UMI. Assessment of UMIs in Hep-G2 and A-549 cells showed no overrepresentation of single UMI groups for each allele, indicating a negligible impact of clonal PCR amplification products on measured DNA methylation levels (Additional file 1: Figures S4–S9).

### Targeted DNA methylation data analysis

The targeted BS data were reviewed and corrected with the fastQC [55] and cutadapt [56] tools for adapter content and sequencing quality. A read was kept for processing when its minimum length was 100 nucleotides and the minimum quality was 25. The sequencing quality values decreased near the end of the reads, as is typical for Illumina sequencing, demonstrating the anticipated accumulation of low sequencing quality scores, particularly in mate 2. A total of 120 nucleotides were automatically removed from the end of mate 2 because they did not meet the strict quality standards (quality scores ≥ 25), which are applied to assure good data quality and reliable base calls. The reads were then aligned against a gene-specific reference (Supplementary Methods) using BISMARK [57] with bowtie2 [58] and the non-directional protocol. Additionally, to account for alignment errors and enable later deconvolution of allele-specific DNA methylation rates, the allele-specific locations were N-masked. The alignments were then divided using SNPsplit [59] which uses the annotated SNPs to discriminate between the two alleles. Finally, DNA methylation calling was performed on the split alignments using BISMARK's methylation extractor function (with the no_overlap and comprehensive parameters). Only the samples with a minimum number of 500 reads after DNA methylation calling were included in further analysis. When calculating the average DNA methylation gain after ASEE, the CpG sites included in the sgRNA binding site were excluded since no DNA methylation can occur at this place. The DNA methylation gain was estimated according to the samples transfected with the scrambled sgRNA, when these were available. Alternatively, the untreated samples were used to compute the difference between treated and reference samples.

At least 40,000 raw reads were obtained for Hep-G2 after NGS and at least 21,000 passed the filters of quality control and were processed for downstream analysis of the *TERT* promoter region covered by BS1 (Fig. 1, Additional file 1: Table S4). At least 70,000 raw reads were acquired for A-549 samples after NGS and at least 13,000 passed the filters of quality control and were processed for downstream analysis of the *TERT* promoter region covered by BS1 and BS2 (Fig. 1, Additional file 1: Table S4). The DNA methylation analysis of the *VEGFA* locus was performed with the same workflow described above, omitting the splitting of the aligned reads into two different alleles. The DNA methylation analysis of BS1 in HEK293 was performed in the same way as the analysis for the *VEGFA* locus since this cell line lacked a *TERT* promoter sequence variant. At least 12,500 raw reads were obtained for HEK293 after NGS and at least 10,300 passed the filters of quality control and were processed for downstream analysis of the *TERT* promoter region covered by BS1 (Fig. 1, Additional file 1: Table S4). The average bisulfite conversion rate was ~ 99% in all analyzed Hep-G2 and HEK293 samples (Additional file 1: Table S4). The average bisulfite conversion rate of A-549 samples was ~ 99% for BS1 and VEGFA assays (Additional file 1: Table S4).

### HTG transcriptome analysis

For functional readout of triple-positive cells, RNA was extracted using the Quick-DNA/RNA™ Microprep Plus Kit (Zymo Research, Irvine, California, USA) according to the manufacturer’s protocol. HTG Transcriptome Panel (2 × 8) assay which covers the vast majority of the human mRNA transcripts including isoforms with 19,616 probes (HTG Molecular Diagnostics, Inc., Tuscon, Arizona, USA) required 70 ng of extracted RNA. After target protection, 4 μL was taken from each sample for library preparation (addition of adapters and molecular barcodes) with the HTG EdgeSeq (Illumina) Tag Pack (HTG Molecular Diagnostics, Inc., Tuscon, Arizona, USA) and the OneTaq® Hot Start 2X Master Mix in GC Buffer (NEB, Ipswich, Massachusetts, USA). The indexing PCR was performed in a Labcycler Basic 011–103 (Sensoquest, Göttingen, Germany) with the following PCR conditions: 4 min at 95 °C, 19 cycles of 15 s at 95 °C, 45 s at 56 °C, 45 s at 68 °C and finally 10 min at 68 °C. After library purification with AMPure XP magnetic beads (Beckman Coulter Life Sciences, Brea, California, USA) according to HTG instructions, the purified libraries were quantified using the KAPA Library Quant Kit (Illumina) Universal qPCR mix (Roche, Basel, Switzerland) and the LightCycler 480 II (Roche, Basel, Switzerland). The libraries were subsequently sequenced with a NextSeq sequencer (Illumina, San Diego, California, USA) using the Illumina NextSeq 500/550 High output v2.5 Reagent Kit (75 cycles) (Illumina, San Diego, California, USA). At least 19,647,280 raw reads were obtained for each sample (Additional file 1: Table S5). Quality control was done using the HTG EdgeSeq Reveal Software (HTG Molecular Diagnostics, Inc., Tuscon, Arizona, USA). Mean and standard deviation of Log2 transformed CPM values were plotted to show gene expression for *TERT* and *VEGFA*. All CPM values used in this study are based on at least three independent experiments and can be found in Additional file 1: Table S5.

### Zombie NIR™ fixable viability experiments on Hep-G2 and A-549 cell lines and flow cytometry analysis

In order to assess the effect of ASEE on cell viability, Hep-G2 were co-transfected using all available *TERT* sgRNAs (C228T-mut, C228T-wt and rs2853669-alt) and the scrambled sgRNA. Cell viability experiments were performed using the Zombie NIR™ Fixable Viability Kit (BioLegend, San Diego, California, USA) following the manufacturer’s instructions. The cells were subsequently analyzed 48, 72 and 96 h post-transfection with the BD LSRFortessa™ Flow Cytometer (BD Biosciences, New Jersey, USA) and the BD FACSDiva™ Software (BD Biosciences, New Jersey, USA). The percentage of dead cells within the triple-positive, the single-positive and triple-negative population was calculated for each sample based on the Zombie staining fluorescence. For the A-549 cell line, co-transfection and cell viability experiments were performed as mentioned above with rs2853669-alt and scrambled sgRNAs. The percentage of dead cells in the triple-positive and triple-negative population was evaluated within each sample by calculating the average and standard deviation based on three technical replicates.

### Statistical analysis

For comparison of DNA methylation between each CpG site among the different samples, a 2-tailed T test for samples with same variance was performed and the P values were Bonferroni corrected. When the q value was > 1, it was considered as 1 for visualization purposes in the respective figures. Corrected P values (q value) lower than 0.05 were considered statistically significant and were shown in the respective figures. All comparisons conducted in this study were done by using the scrambled sgRNA transfected samples as reference when these were available. Otherwise, untreated samples were taken as reference. For comparison between time points in the viability experiments, a 2-tailed T test for samples with same variance was performed.

### Supplementary Information

**Additional file 1. **Supplementary information. This file contains the reference sequences used for the targeted DNA methylation analyses of the *TERT* and *VEGFA* loci as well as information about the SNV positions used to distinguish the DNA methylation calls between the alleles. Additionally, it includes the Supplementary Figures 1–9 and the Supplementary Tables 1-5 with their respective legends.

## Data Availability

All data analyzed during this study are included in this published article and its supplementary information files.
