# Supplementary material for: Targeting oncogenic TERT promoter variants by allele-specific epigenome editing
Source: Clin Epigenetics. 2023 Nov 22;15:183. doi: 10.1186/s13148-023-01599-2 (PMC10666398; doi:10.1186/s13148-023-01599-2)
Supplement: Supplementary file 1 — Additional file 1. Supplementary information. This file contains the reference sequences used for the targeted DNA methylation analyses of the TERT and VEGFA loci as well as information about the SNV positions used to distinguish the DNA methylation calls between the alleles. Additionally, it includes the Supplementary Figures 1–9 and the Supplementary Tables 1-5 with their respective legends. [file 13148_2023_1599_MOESM1_ESM.docx]

**SUPPLEMENTARY INFORMATION**

**Reference sequences used for DNA methylation analysis**

***TERT* BS1**

>hg19_dna range=chr5:1,295,112-1,295,401

NNNNNNNNNNGTGGCCGGGGCCAGGGCTTCCCACGTGCGCAGCAGGACGCAGCGCTGCCTGAAACTCGCGCCGCGAGGAGAGGGCGGGGCCGCGGAAAGGAAGGGGAGGGGCTGGGAGGGCCCGGANGGGGCTGGGCCGGGGACCCGGGAGGGGTCGGGACGGGGCGGGGTCCGCGCGGAGGAGGCGGAGCTGGAAGGTGAAGGGGCAGGACGGGTGCCCGGGTCCCCAGTCCCTCCGCCACGTGGGNAGCGCGGTCCTGGGCGTCTGTGCCCGCGAATCCACTGGGAGCCCGGCCTGGCNNNNNNNNNN

The following positions (shown in the table below and highlighted in red in the sequence above) were N-masked in order to perform allele-specific DNA methylation analysis. The “N” letters framing each reference sequence were added to facilitate efficient alignment of the reads throughout the whole reference sequence independent of the adapters and UMIs that are added at the primer sequences.

| Genome | Variant name | BS assay position | Number of affected nucleotides | Observed nucleotides |
| --- | --- | --- | --- | --- |
| hg19 | C228T | 127 | 1 | G/A |
| hg19 | rs2853669 | 248 | 1 | A/G |

***TERT* BS2**

>hg19_dna range=chr5:1,295,290-1,295,642

NNNNNNNNNNAGTTGGAAGGTGAAGGGGTAGGACGGGTGTTCGGGTTTTTAGTTTTTTCGTTACGTGGGNAGCGCGGTTTTGGGCGTTTGTGTTCGCGAATTTATTGGGAGTTCGGTTTGGTTTCGATAGCGTAGTTGTTTCGGGCGGATTCGGGGGTTTGGGTCGCGTTTTTTCGTTCGCGCGTCGTTCGCGTTTTTAGGGTGTAGGGACGTTAGCGAGGGTTTTAGCGGAGAGAGGTCGAATCGGTTTAGGTTGTGGGGTAATTCGAGGGAGGGGTTATGATGTGGAGGTTTTGGGAATAGGTGCGTGCGGCGATTTTTTGGTCGTTGGTTTGATTCGGAGATTTAGGGTTGTTTTTAGGTNNNNNNNNNN

The following positions (shown in the table below and highlighted in red in the sequence above) were N-masked in order to perform allele-specific DNA methylation analysis. The “N” letters framing each reference sequence were added to facilitate efficient alignment of the reads throughout the whole reference sequence independent of the adapters and UMIs that are added at the primer sequences.

| Genome | Variant name | BS assay position | Number of affected nucleotides | Observed nucleotides |
| --- | --- | --- | --- | --- |
| hg19 | rs2853669 | 70 | 1 | A/G |

***VEGFA***

> hg19_dna range=chr6:43,738,171-43,738,372

NNNNNNNNNNGCTTGCCATTCCCCACTTGAATCGGGCCGACGGCTTGGGGAGATTGCTCTACTTCCCCAAATCACTGTGGATTTTGGAAACCAGCAGAAAGAGGAAAGAGGTAGCAAGAGCTCCAGAGAGAAGTCGAGGAAGAGAGAGACGGGGTCAGAGAGAGCGCGCGGGCGTGCGAGCAGCGAAAGCGACAGGGGCAAAGTGAGTGACCNNNNNNNNNN

The “N” letters framing each reference sequence were added to facilitate efficient alignment of the reads throughout the whole reference sequence independent of the adapters and UMIs that are added at the primer sequences.

**SUPPLEMENTARY FIGURES**

**
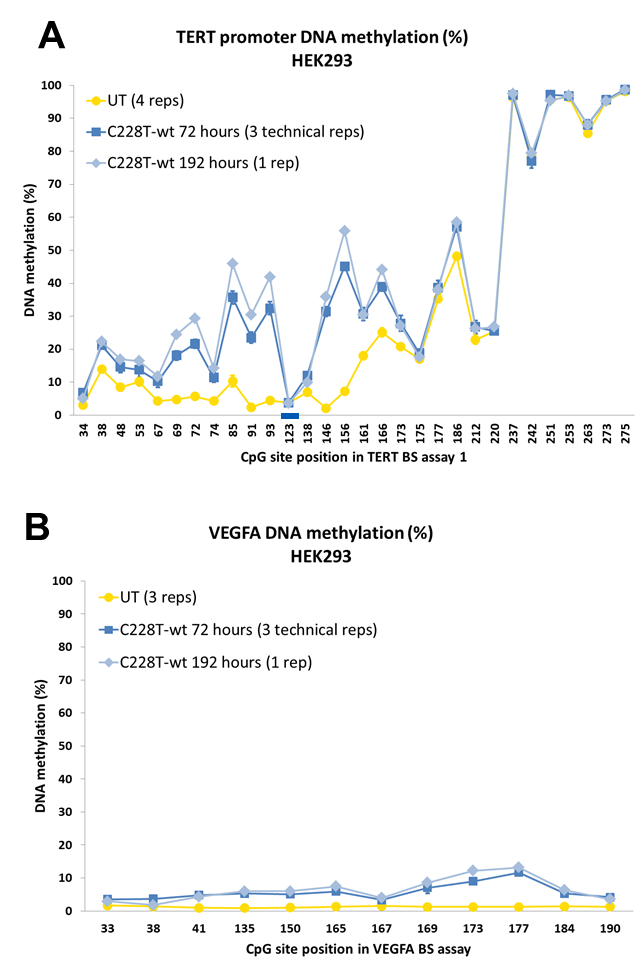
**

**Supplementary Figure 1: *TERT* promoter DNA methylation in HEK293 cells after locus-specific epigenome editing using *TERT* sgRNA targeting the wildtype allele at C228T position 72- and 192-hours post transfection.** Average DNA methylation at *TERT* (A) and *VEGFA* (B) promoter (used as off-target methylation control) after locus-specific epigenome editing. The DNA methylation is shown 72 hours (dark blue) and 192 hours (light blue) post transfection with the sgRNA that targets the wildtype allele (C228T-wt, which in HEK293 cells is the only present allele) at C228T position. (A, B) Untreated HEK293 cells (UT) are shown in yellow. X-axis shows the position of CpG sites in the BS1 (A) and *VEGFA* (B) assay according to the reference sequence used in the analysis. (A) The blue line under position 123 shows the sgRNA binding site.

**
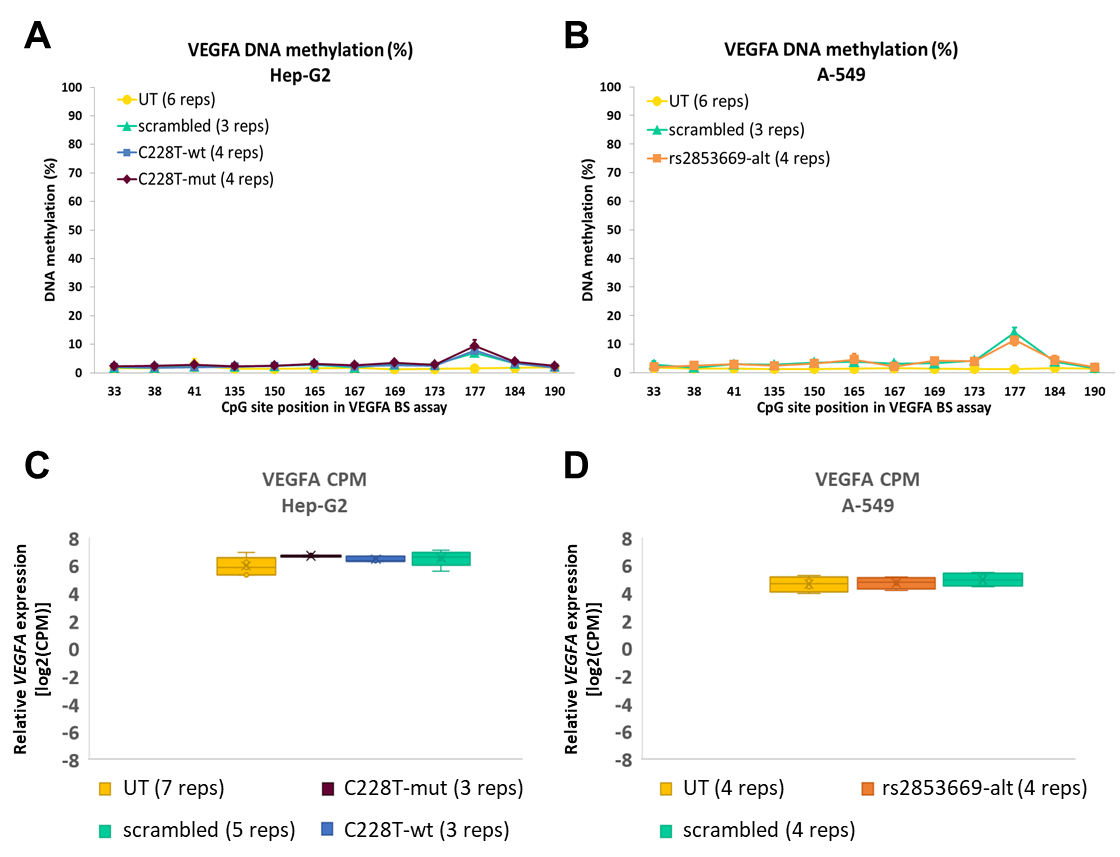
**

**Supplementary Figure 2: *VEGFA* promoter DNA methylation and RNA expression in Hep-G2 and A-549 cells after ASEE at the *TERT* promoter.** Average DNA methylation at *VEGFA* promoter region after ASEE with different sgRNAs in Hep-G2 (A) and A-549 (B). (A, B) X-axis shows the position of CpG sites in the assay according to the reference sequence used in the analysis. *VEGFA* RNA expression shown as log2 transformed counts per million (CPM) values in Hep-G2 (C) and A-549 (D) transfected with different sgRNAs. Yellow color corresponds to untreated cells (UT), purple and blue color corresponds to cells transfected with *TERT* sgRNA targeting the mutated (C228T-mut) and wildtype (C228T-wt) allele at C228T position respectively, light green color corresponds to cells transfected with scrambled sgRNA and orange color corresponds to cells transfected with *TERT* sgRNA targeting the G alternative allele at rs2853669 SNP position (rs2853669-alt).


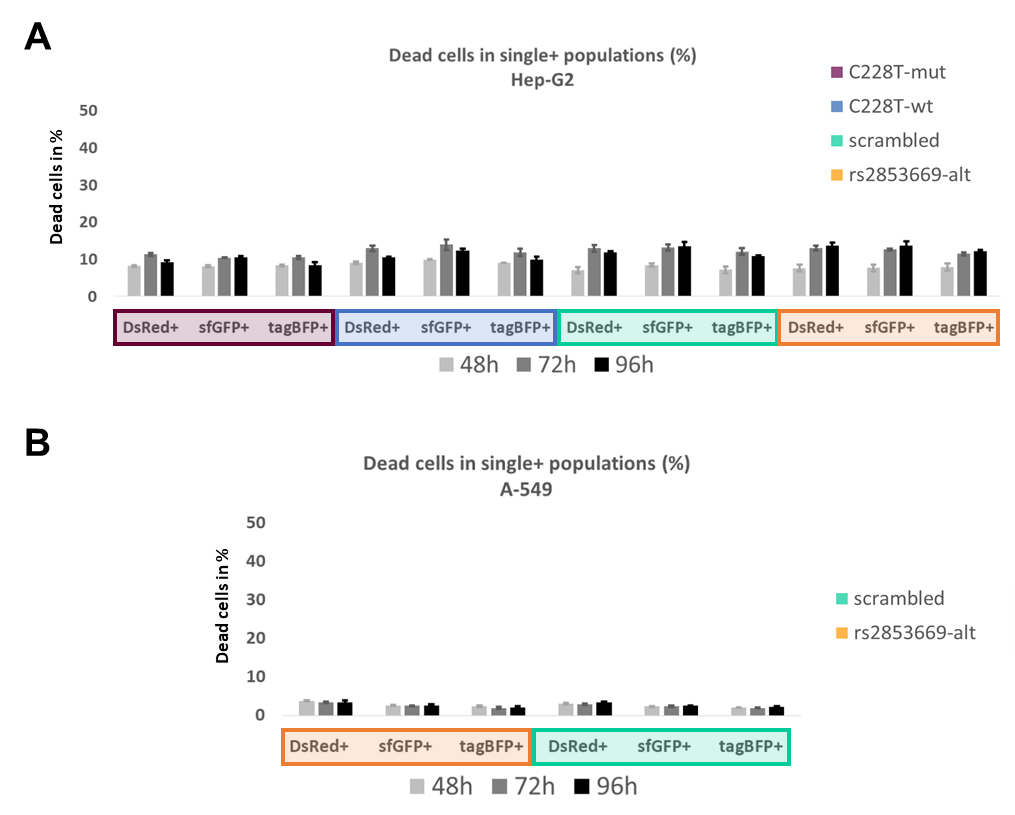


**Supplementary Figure 3: Viability of Hep-G2 and A-549 single-positive populations after transfection with the 3-plasmid system.** Bar plots showing the percentage (%) of dead cells within the Hep-G2 single-positive populations (A) and A-549 single-positive populations (B) carrying only one component (dCas9-10X Suntag vector – tagBFP, DNMT3A-3L vector – sfGFP, or sgRNA vector – DsRed) of the 3-plasmid system. Boxes underneath the x-axis indicate the different experiments conducted in each cell line. (A) Purple and blue box corresponds to single-positive populations among the Hep-G2 cell transfected with the 3-plasmid system and the TERT sgRNA targeting the mutated (C228T-mut) and wildtype allele (C228T-wt) at the C228T mutation position respectively. Light green and orange boxes correspond to the single-positive populations among the cells transfected with the 3-plasmid system and the TERT sgRNA targeting the alternative G allele at rs2853669 (rs2853669-alt) and scrambled sgRNA respectively (A, B).


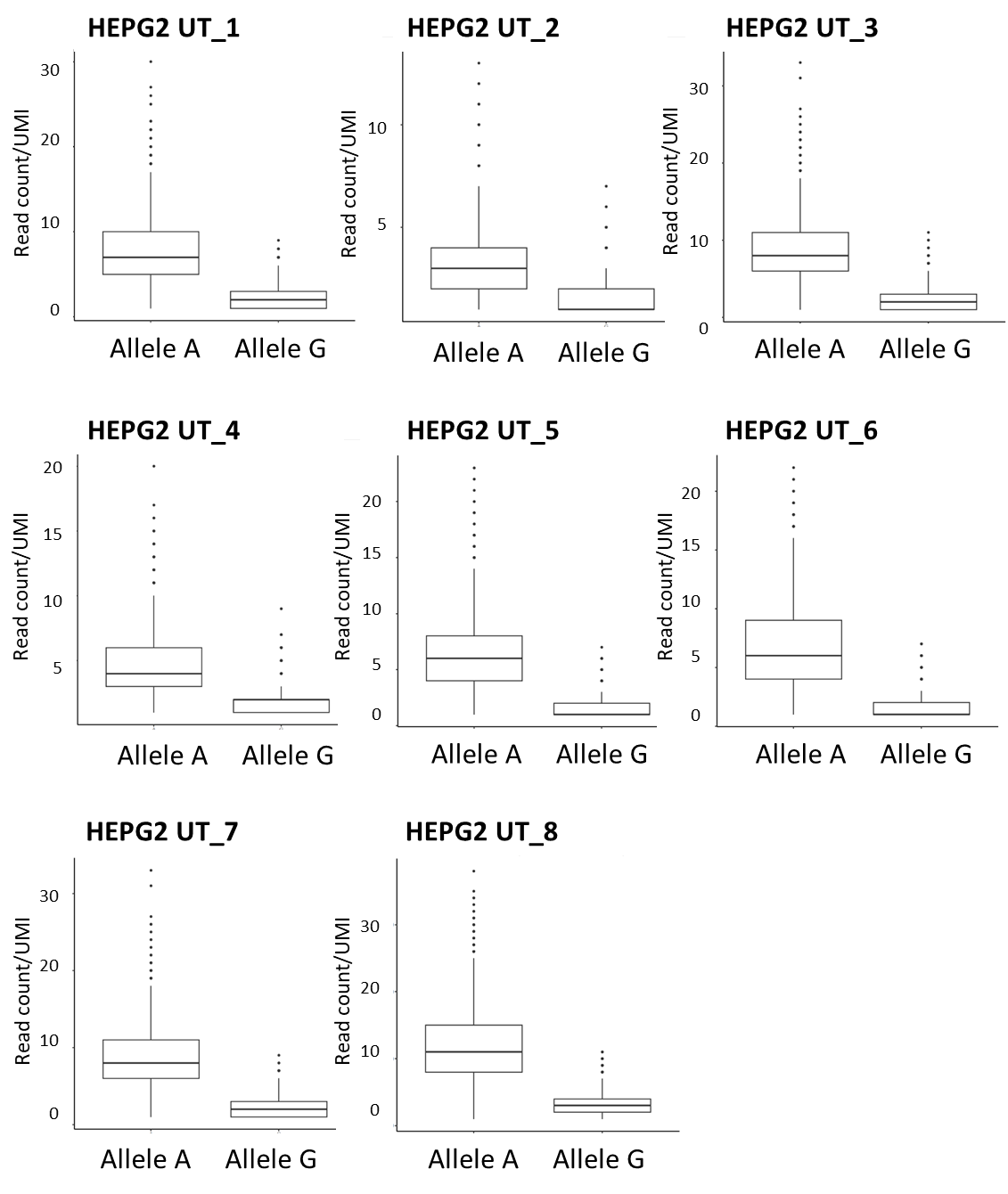


**Supplementary Figure 4: Allelic UMI frequency in the untreated Hep-G2 samples (UT) used in this study.** Boxplots indicating the number of reads per UMI group for the two alleles of the C228T mutation in eight independent replicates in Hep-G2. The targeted alleles for C228T correspond to the opposite strands since the PAM site for dCas9 lies on the opposite strand of this mutation. Thus, the A allele corresponds to the mutated allele and the G allele corresponds to the wildtype allele.


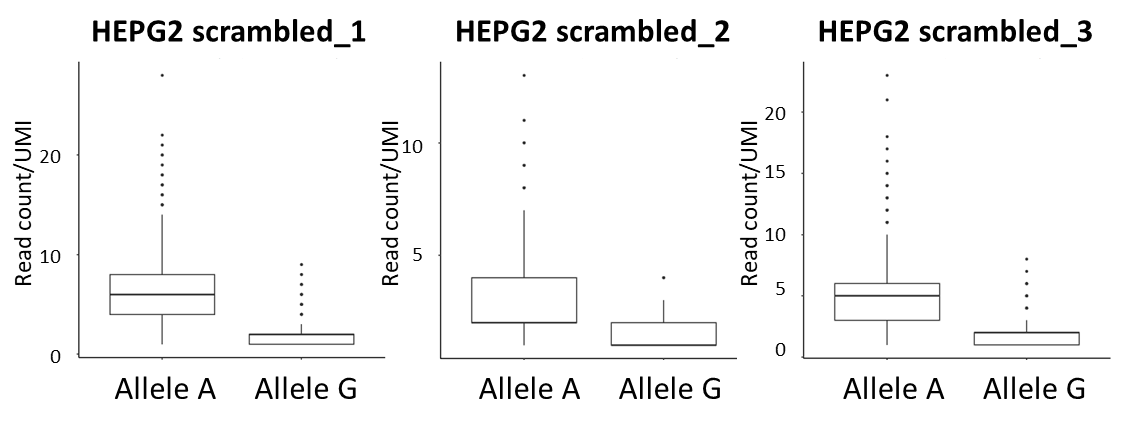


**Supplementary Figure 5: Allelic UMI frequency in the Hep-G2 samples that were transfected with the scrambled sgRNA.** Boxplots indicating the number of reads per UMI group for the two alleles of the C228T mutation in Hep-G2 in three replicates. The targeted alleles for C228T correspond to the opposite strands since the PAM site for dCas9 lies on the opposite strand of this mutation. Thus, the A allele corresponds to the mutated allele and the G allele corresponds to the wildtype allele.


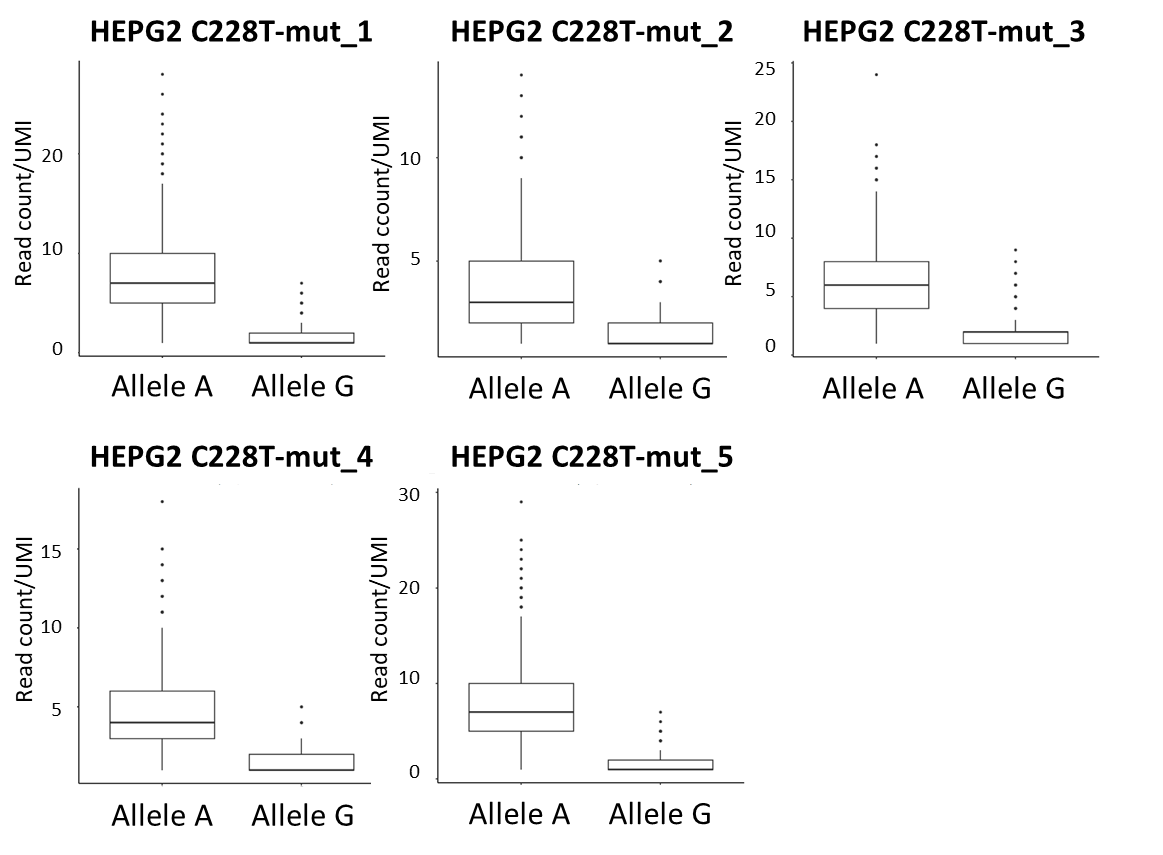


**Supplementary Figure 6: Allelic UMI frequency in the Hep-G2 samples that were transfected with the C228T-mut vector which targets the mutated allele of the C228T mutation.** Boxplots indicating the number of reads per UMI group for the two alleles of the C228T mutation in Hep-G2 in five replicates. The targeted alleles for C228T correspond to the opposite strands since the PAM site for dCas9 lies on the opposite strand of this mutation. Thus, the A allele corresponds to the mutated allele and the G allele corresponds to the wildtype allele.


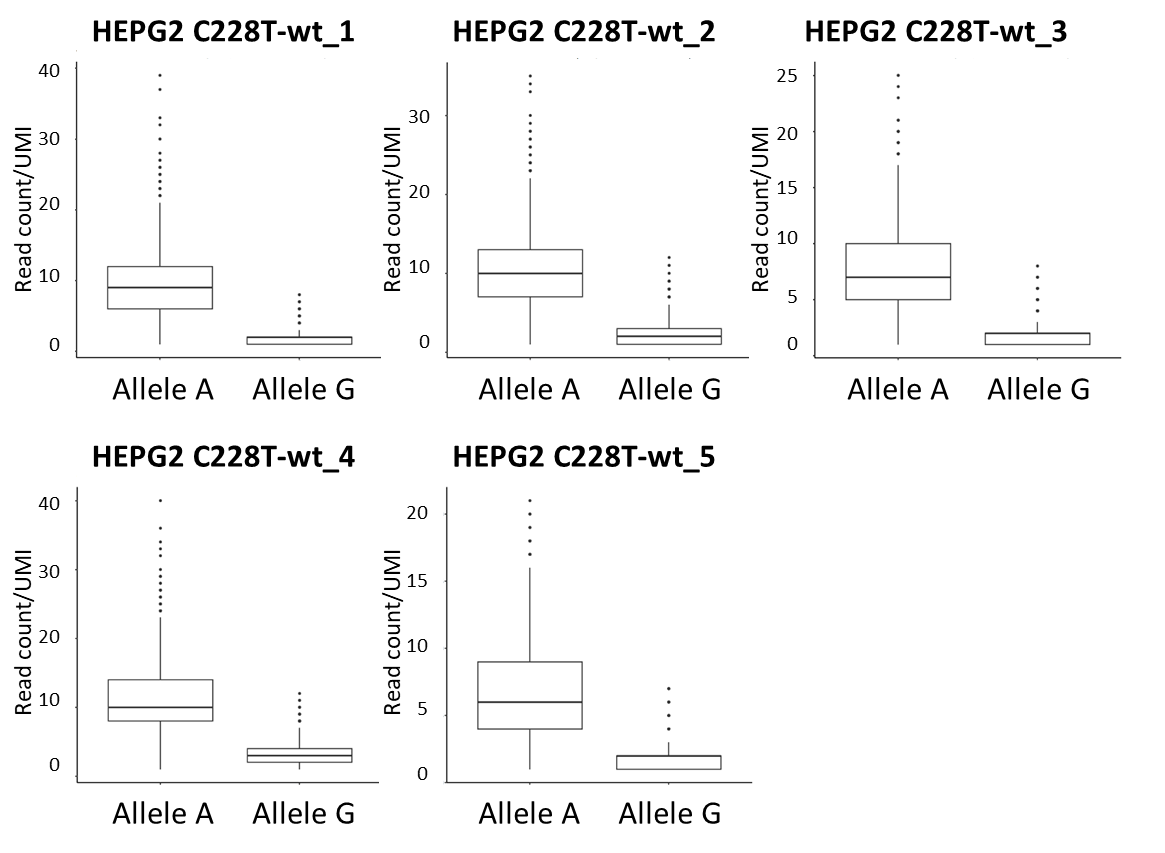


**Supplementary Figure 7: Allelic UMI frequency in the Hep-G2 samples that were transfected with the C228T-wt vector which targets the wildtype allele of the C228T mutation.** Boxplots indicating the number of reads per UMI group for the two alleles of the C228T mutation in Hep-G2 in five replicates. The targeted alleles for C228T correspond to the opposite strands since the PAM site for dCas9 lies on the opposite strand of this mutation. Thus, the A allele corresponds to the mutated allele and the G allele corresponds to the wildtype allele.


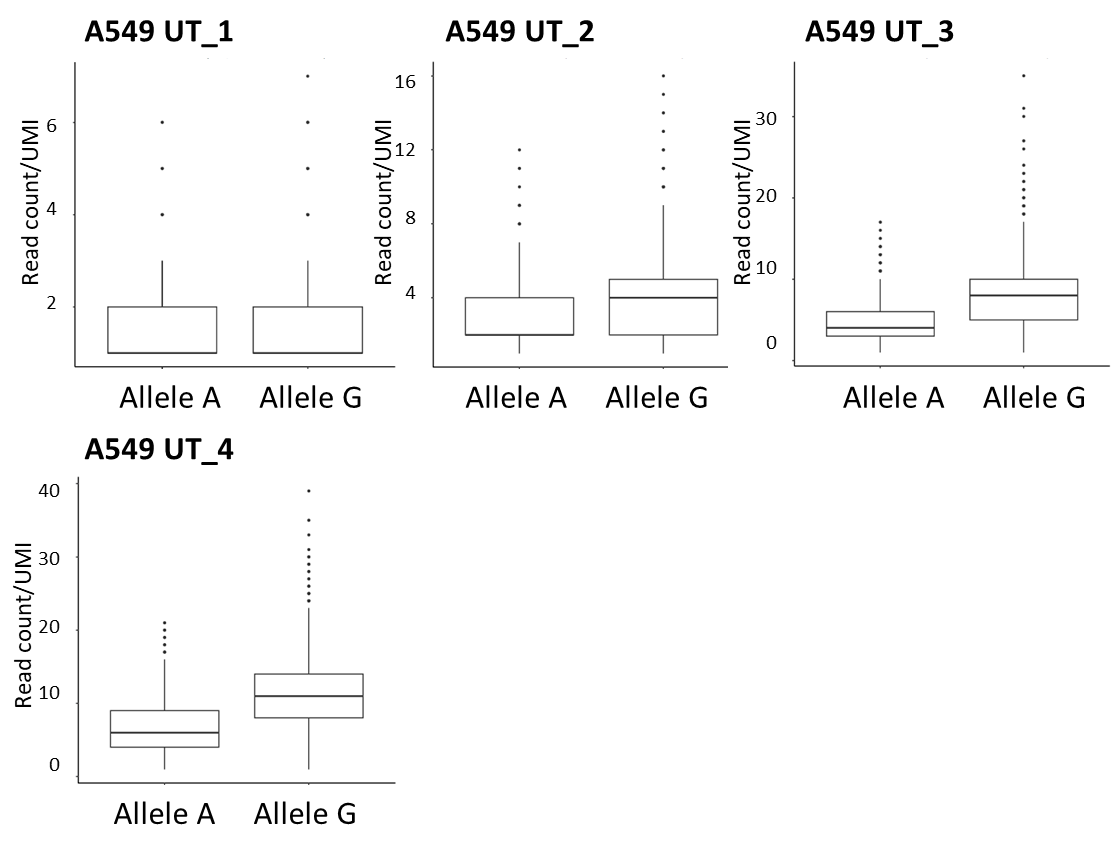


**Supplementary Figure 8: Allelic UMI frequency in the untreated A-549 samples (UT) used in this study.** Boxplots indicating the number of reads per UMI group for the two alleles of the rs2853669 SNP (A: reference, G: alternative) in A-549 in four replicates.


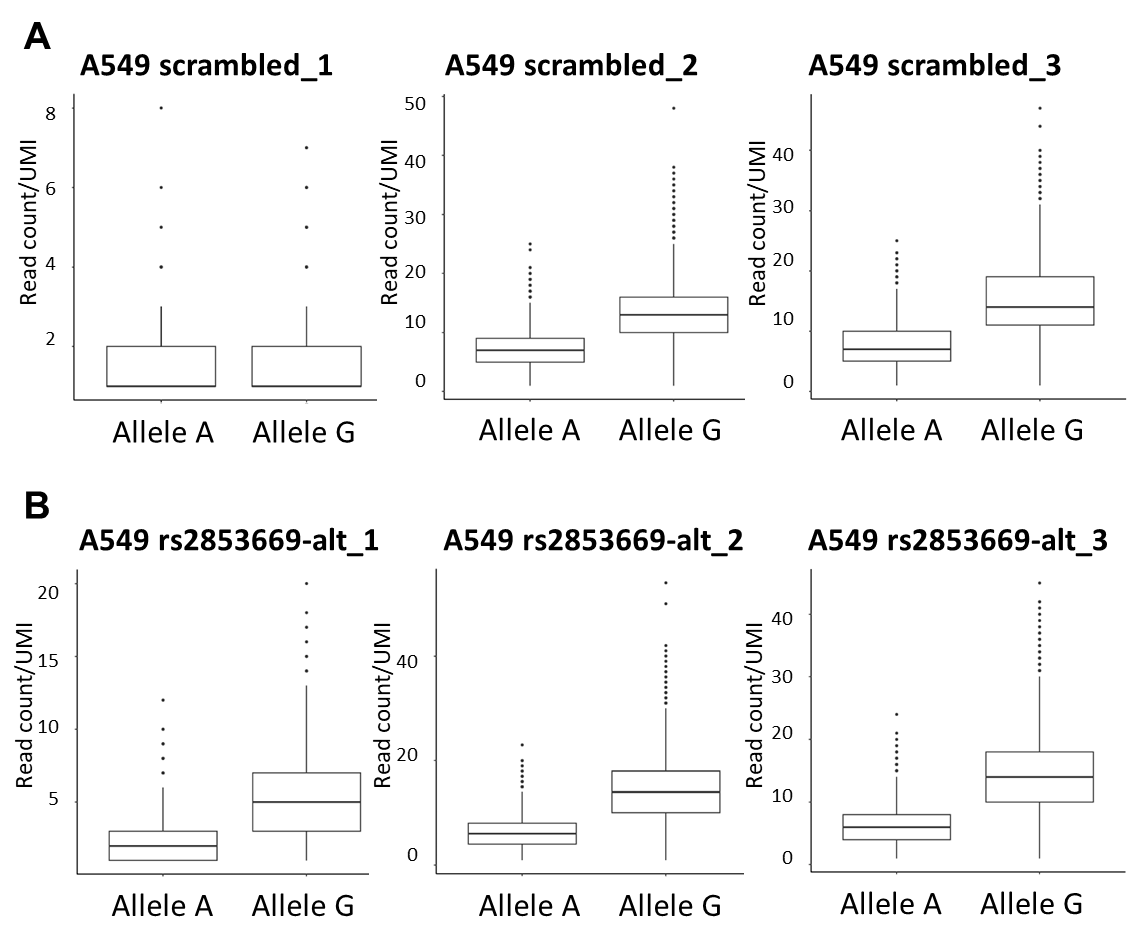


**Supplementary Figure 9: Allelic UMI frequency in the A-549 samples that were transfected with the scrambled sgRNA and the sgRNA targeting the variant allele of rs2853669.** A) Boxplots indicating the number of reads per UMI group for the two alleles of the rs2853669 SNP after transfection with the scrambled sgRNA in A-549 (three replicates). B) Boxplots indicating the number of reads per UMI group for the two alleles of the rs2853669 SNP (A: reference, G: alternative) after transfecting A-549 cells with the sgRNA rs2853669-alt that targets the variant allele (three replicates).

**SUPPLEMENTARY TABLES**

**Supplementary Table 1:***TERT* promoter SNVs detected by Sanger and bisulfite sequencing. Zygosity of rs2853669 is indicated in the third column. Cell lines with no data (NA) did not fulfill the quality requirements and were excluded from further analysis.

| **Cell line name** | **SNVs detected by Sanger sequencing using SeqScanner2** | **Zygosity of SNVs** |
| --- | --- | --- |
| Daudi | rs2853669 (A>G) | heterozygous |
| Raji | wt |  |
| BL-41 | wt |  |
| BL-70 | rs35226131 (C>T) , rs35161420 (C>G) | heterozygous |
| BLUE-1 | NA |  |
| CA46 | rs2853669 (A>G) | heterozygous |
| DG-75 | rs2853669 (A>G) | heterozygous |
| EB1 | rs2853669 (A>G) | heterozygous |
| Namalwa | wt |  |
| BL-2 | wt |  |
| Ramos | wt |  |
| JI | wt |  |
| EB3 | wt |  |
| MC116 | rs2853669 (A>G) , rs907929058 (C>T) | heterozygous |
| U-698-M | rs35226131 (C>T) | heterozygous |
| Karpas-422 | wt |  |
| SU-DHL-10 | rs2853669 (A>G) | heterozygous |
| SU-DHL-6 | NA |  |
| RIVA | wt |  |
| OCI-Ly3 | rs2853669 (A>G) | heterozygous |
| U-2932R1 | rs35226131 (C>T) | heterozygous |
| U-2932R2 | rs35226131 (C>T) | heterozygous |
| HDLM-2 | rs2853669 (G) | homozygous |
| L-1236 | wt |  |
| L-428 | rs1384474222 (C>T) , rs2853669 (A>G) | heterozygous |
| L-540 | rs2853669 (G) | homozygous |
| SUP-HD1 | rs2853669 (G) | homozygous |
| L-591 | wt |  |
| U-HO1 | rs2853669 (A>G) | heterozygous |
| KM-H2 | wt |  |
| AM-HLH | rs2853669 (A>G) | heterozygous |
| HD-70 | wt |  |
| SU-DHL-5 | rs2853669 (A>G) | heterozygous |
| HT | rs2853669 (A>G) | heterozygous |
| MLMA | wt |  |
| LM-1 | rs2853669 (A>G) | heterozygous |
| BJAB | wt |  |
| WW1-LCL | rs2853669 (G) | homozygous |
| GM12878 | rs35550267 (G>A) | heterozygous |
| IARC-304 | wt |  |
| DEV | rs2853669 (A>G) | heterozygous |
| CRO-AP3 | rs2853669 (A>G) | heterozygous |
| HH-B2 | rs2853669 (A>G) | heterozygous |
| A-549 | rs2853669 (A>G) | heterozygous |
| HEK293 | wt |  |
| Hep-G2 | C228T , rs2853669 (A>G) | heterozygous |
| BALM-18 | rs2853669 (A>G) | heterozygous |
| BC-1 | rs2853669 (G) | homozygous |
| BC-2 | rs35226131 (C>T) , rs35161420 (C>G) | heterozygous |
| BC-3 | NA |  |
| BL-30 | rs2853669 (A>G) | heterozygous |
| CCRF-CEM | wt |  |
| CRO-AP2 | NA |  |
| CRO-AP5 | NA |  |
| CRO-AP6 | NA |  |
| Farage | rs2853669 (G) | homozygous |
| HBL-1 | wt |  |
| Hep 3B2.1-7 | wt |  |
| HH | rs2853669 (A>G) | heterozygous |
| Huh-7 | NA |  |
| HuT 78 | rs2853669 (A>G) | heterozygous |
| Jurkat | wt |  |
| Karpas-231 | wt |  |
| Karpas-299 | wt |  |
| Karpas-353 | rs2853669 (A>G) | heterozygous |
| KE-37 | rs2853669 (A>G) | heterozygous |
| KIS-1 | wt |  |
| KOPN-8 | rs2853669 (G) | homozygous |
| LNCaP | NA |  |
| Ly11212 | wt |  |
| Ly12318 | rs2853669 (A>G) | heterozygous |
| Ly12657 | wt |  |
| Ly13136 | wt |  |
| MHH-PREB-1 | wt |  |
| NALM-33 | rs2853669 (A>G) | heterozygous |
| OCI-Ly1 | rs2853669 (A>G) | heterozygous |
| OCI-Ly10 | wt |  |
| Phoenix-Ampho | NA |  |
| RC-K8 | wt |  |
| Reh | C>G at SNP rs1561215593 (which is C>T transition) | heterozygous |
| RS4;11 | wt |  |
| SK-MM-1 | wt |  |
| SU-DHL-1 | wt |  |
| SUP-T11 | NA |  |
| THP-1 | wt |  |
| TK | wt |  |
| U-2932 | wt |  |

**Supplementary Table 2:** Vector information used in transient transfection experiments. For Addgene plasmids, the Addgene number is given in the first column. The targeted alleles for C228T correspond to the opposite strands since the PAM site for dCas lies on the opposite strand of this mutation.

| **Addgene** | **Name** | **Size (bp)** | **Cell line** | **Target allele** | **Target position (hg19)** | **Fluorphore used for FACS** | **Bacerial resistance** | **sgRNA sequence** |
| --- | --- | --- | --- | --- | --- | --- | --- | --- |
| #174141 | dCas9-10X Suntag | 10,032 | A-549 and Hep-G2 |  |  | TagBFP | Ampicillin |  |
| #154141 | DNMT3A R887E-3L | 6,200 | A-549 and Hep-G2 |  |  | Superfolder GFP (sfGFP) | Ampicillin |  |
| - | C228T-mut | 5,097 | Hep-G2 | A | chr5:1,295,228: C>T | DsRed-Monomer | Kanamycin | GGGGCTGGGAGGGCCCGGAA |
| - | C228T-wt | 5,097 | HEK293 and Hep-G2 | G | chr5:1,295,228: C>T | DsRed-Monomer | Kanamycin | GAGGGGCTGGGAGGGCCCGG |
| - | scrambled sgRNA | 5,085 | A-549 and Hep-G2 |  |  | DsRed-Monomer | Kanamycin | GAACAGTCGCGTTTGCGACT |
| - | rs2853669-alt | 5,097 | A-549 and Hep-G2 | G | rs2853669: A>G | DsRed-Monomer | Kanamycin | CCCAGTCCCTCCGCCACGTG |

**Supplementary Table 3:** Primer sequences used in this study. For Sanger sequencing primers, underlined letters indicate the Universal Sequences (US) used for sequencing and the rest is locus-specific sequence. For BS primers, capital letters indicate the overhang adapters used to attach the Illumina elements such as: Flow cell binding regions for clustering, sequencing primer binding region and indexes unique for each sample. Small letters indicate the *TERT* locus-specific sequences used to amplify the region that was studied by Bisulfite sequencing and NNNNNN in the forward primer sequence indicate the UMI sequences. Primers were ordered from biomers.net (Ulm, Germany).

| **Assay** | **Primer Name** | **Primer Sequence** | **PCR product Size** | **PCR product position (hg19)** |
| --- | --- | --- | --- | --- |
| TERT prom Sanger sequencing | TERT-prom-US1 | gtagcgcgacggccagtgattcgacctctctccgctg | 516 bp | chr5:1,295,043-1,295,524 |
| TERT prom Sanger sequencing | TERT-prom-US2 | cagggcgcagcgatgacacctcgcggtagtggctg |  |  |
| Targeted BS (NGS) | TERT_BS_16S_fw_overhang | TCGTCGGCAGCGTCAGATGTGTATAAGAGACAGNNNNNNgtggtyggggttagggtttt | 363 bp | chr5:1,295,112-1,295,401 |
| Targeted BS (NGS) | TERT_BS_16S_rev_overhang | GTCTCGTGGGCTCGGAGATGTGTATAAGAGACAGaccaaaccraactcccaataaa |  |  |
| Targeted BS (NGS) | VEGFA_BS_Nextera_fw_overhang_umi | TCGTCGGCAGCGTCAGATGTGTATAAGAGACAGNNNNNNgtttgttattttttatttgaat | 275 bp | chr6:43,738,171-43,738,372 |
| Targeted BS (NGS) | VEGFA_BS_Nextera_rev_overhang | GTCTCGTGGGCTCGGAGATGTGTATAAGAGACAGaatcactcactttacccctatc |  |  |
| Targeted BS (NGS) | TERT_BS2_fw_overhang_umi | TCGTCGGCAGCGTCAGATGTGTATAAGAGACAGNNNNNNagttggaaggtgaagggg | 426 bp | chr5:1,295,290-1,295,642 |
| Targeted BS (NGS) | TERT_BS2_rev_overhang | GTCTCGTGGGCTCGGAGATGTGTATAAGAGACAGacctaaaaacaaccctaaatctcc |  |  |
| dCas9 Plasmid validation | PL322_F1 | gattcattaatgcagctgtg | 700 bp |  |
| dCas9 Plasmid validation | PL322_R1 | gttgctgaagatctcttgc |  |  |
| DNMT3A-3L Plasmid validation | PL323_F1 | catccactgtgaatgataagc | 569 bp |  |
| DNMT3A-3L Plasmid validation | PL323_R1 | atttgtgacatcttccacg |  |  |
| sgRNA Plasmid validation | PL324-330_sgRNA_F1 | ggactatcatatgcttaccgtaac | 512 bp |  |
| sgRNA Plasmid validation | PL324-330_sgRNA_R1 | cttgattagggtgatggttc |  |  |

**Supplementary Table 4:** Table containing information on total read counts, read counts after trimming (QC reads) and bisulfite conversion rates for all BS samples and all different regions studied with BS. Bisulfite conversion rates calculated using the following formula: (T reads / [T reads+ C reads]), where T and C reads correspond to read counts in positions outside of CpG context.

|  | **TERT BS1** | | | **TERT BS2** | | | **VEGFA** | | |
| --- | --- | --- | --- | --- | --- | --- | --- | --- | --- |
| Sample name | Total read count | QC reads | Bisulfite conversion rate (%) | Total read count | QC reads | Bisulfite conversion rate (%) | Total read count | QC reads | Bisulfite conversion rate (%) |
| HEPG2 UT_1 | 54,058 | 21,397 | 98.9 | NA | NA | NA | NA | NA | NA |
| HEPG2 UT_2 | 55,824 | 35,117 | 98.8 | NA | NA | NA | NA | NA | NA |
| HEPG2 UT_3 | 75,869 | 64,488 | 98.7 | NA | NA | NA | 68,226 | 67,686 | 98.8 |
| HEPG2 UT_4 | 82,970 | 72,850 | 98.7 | NA | NA | NA | 119,336 | 118,199 | 98.8 |
| HEPG2 UT_5 | 141,939 | 49,970 | 98.9 | NA | NA | NA | 101,159 | 97,443 | 99.8 |
| HEPG2 UT_6 | 205,871 | 53,830 | 99.2 | NA | NA | NA | 192,316 | 190,403 | 99.1 |
| HEPG2 UT_7 | 83,878 | 64,340 | 98.4 | NA | NA | NA | 166,904 | 165,244 | 98.9 |
| HEPG2 UT_8 | 119,957 | 89,370 | 98.1 | NA | NA | NA | 28,967 | 28,653 | 98.2 |
| HEPG2 C228T-wt_1 | 57,818 | 52,347 | 98.7 | NA | NA | NA | 1,091 | 1,054 | 98.9 |
| HEPG2 C228T-wt_2 | 123,323 | 91,323 | 98.9 | NA | NA | NA | NA | NA | NA |
| HEPG2 C228T-wt_3 | 84,519 | 72,737 | 98.7 | NA | NA | NA | 94,277 | 93,570 | 98.7 |
| HEPG2 C228T-wt_4 | 134,202 | 106,477 | 99.0 | NA | NA | NA | 273,068 | 269,837 | 98.7 |
| HEPG2 C228T-wt_5 | 139,698 | 62,698 | 98.8 | NA | NA | NA | 150,748 | 148,585 | 98.8 |
| HEPG2 C228T-mut_1 | 40,154 | 25,753 | 98.7 | NA | NA | NA | NA | NA | NA |
| HEPG2 C228T-mut_2 | 50,743 | 45,791 | 99.5 | NA | NA | NA | 12,022 | 11,508 | 99.1 |
| HEPG2 C228T-mut_3 | 66,652 | 57,167 | 98.7 | NA | NA | NA | 91,976 | 91,349 | 98.7 |
| HEPG2 C228T-mut_4 | 360,803 | 39,249 | 99.0 | NA | NA | NA | 179,934 | 177,601 | 99.0 |
| HEPG2 C228T-mut_5 | 65,361 | 57,641 | 98.1 | NA | NA | NA | 70,744 | 70,244 | 98.1 |
| HEPG2 scrambled_1 | 64,859 | 54,058 | 98.4 | NA | NA | NA | 104,391 | 103,577 | 98.6 |
| HEPG2 scrambled_2 | 270,261 | 32,252 | 99.1 | NA | NA | NA | 2,075,218 | 2,054,821 | 99.0 |
| HEPG2 scrambled_3 | NA | NA | NA | NA | NA | NA | 364,101 | 348,873 | 99.2 |
| HEPG2 scrambled_4 | 71,883 | 37,330 | 92.4 | NA | NA | NA | NA | NA | NA |
| A549 UT_1 | 415,782 | 13,938 | 99.3 | 81,942 | 75,797 | 89.3 | 65,879 | 65,041 | 99.1 |
| A549 UT_2 | 194,969 | 64,901 | 98.5 | 80,620 | 76,992 | 80.4 | 57,531 | 56,710 | 98.4 |
| A549 UT_3 | 211,760 | 114,209 | 98.9 | 72,870 | 69,856 | 92.9 | 52,381 | 51,668 | 98.8 |
| A549 UT_4 | NA | NA | NA | NA | NA | NA | 35,473 | 32,735 | 99.3 |
| A549 UT_5 | NA | NA | NA | NA | NA | NA | 55,467 | 52,368 | 99.3 |
| A549 UT_6 | 205,919 | 112,612 | 99.0 | 155,498 | 148,404 | 89.5 | NA | NA | NA |
| A549 UT_7 | NA | NA | NA | 103,850 | 95,538 | 81.8 | 473,028 | 463,419 | 99.6 |
| A549 rs2853669-alt_1 | 186,886 | 67,963 | 99.2 | 78,206 | 74,667 | 80.0 | 54,239 | 53,491 | 99.1 |
| A549 rs2853669-alt_2 | NA | NA | NA | NA | NA | NA | 37,588 | 37,017 | 98.3 |
| A549 rs2853669-alt_3 | NA | NA | NA | NA | NA | NA | 65,253 | 64,341 | 99.1 |
| A549 rs2853669-alt_4 | NA | NA | NA | NA | NA | NA | 114,112 | 112,786 | 97.6 |
| A549 rs2853669-alt_5 | 180,068 | 125,831 | 98.7 | 196,716 | 190,264 | 82.5 | NA | NA | NA |
| A549 rs2853669-alt_6 | 197,250 | 129,908 | 98.8 | 185,474 | 178,108 | 92.4 | NA | NA | NA |
| A549 scrambled_1 | 70,788 | 15,806 | 99.2 | NA | NA | NA | 70,180 | 67,148 | 99.3 |
| A549 scrambled_2 | NA | NA | NA | NA | NA | NA | 66,830 | 63,779 | 99.3 |
| A549 scrambled_3 | NA | NA | NA | NA | NA | NA | 59,283 | 56,350 | 99.2 |
| A549 scrambled_4 | 216,740 | 128,291 | 99.0 | 155,851 | 149,352 | 92.3 | NA | NA | NA |
| A549 scrambled_5 | 184,500 | 141,957 | 98.8 | 160,486 | 154,176 | 91.5 | NA | NA | NA |
| A549 scrambled_6 | NA | NA | NA | 280,313 | 251,454 | 86.5 | NA | NA | NA |
| HEK293_UT_1 | 21,815 | 11,945 | 98.3 | NA | NA | NA | 65,684 | 64,741 | 98.7 |
| HEK293_UT_2 | 13,067 | 8,882 | 99.1 | NA | NA | NA | NA | NA | NA |
| HEK293_UT_3 | 745,608 | 74,092 | 99.2 | NA | NA | NA | 264,606 | 261,987 | 99.3 |
| HEK293_UT_4 | 169,888 | 136,832 | 99.6 | NA | NA | NA | 399,060 | 394,441 | 99.6 |
| HEK293_C228T-wt_1 | 17,054 | 14,282 | 98.6 | NA | NA | NA | 52,575 | 51,640 | 98.5 |
| HEK293_C228T-wt_2 | 13,361 | 11,223 | 98.5 | NA | NA | NA | 62,134 | 61,261 | 98.7 |
| HEK293_C228T-wt_3 | 12,525 | 10,372 | 98.5 | NA | NA | NA | 53,075 | 52,405 | 98.7 |
| HEK293_C228T-wt_day8 | 272,178 | 204,472 | 99.2 | NA | NA | NA | 376,174 | 372,586 | 99.4 |

**Supplementary Table 5:**Read count and log2 transformed CPM values that were used in this study after HTG run. The "Group" column contains group information for Hep-G2 and A-549 cell lines. UT corresponds to the untreated cells, C228T-mut and C228T-wt corresponds to the sgRNA that targets the mutated and wildtype allele at the C228T position in Hep-G2 respectively, rs2853669-alt corresponds to the sgRNA that targets the alternative G allele of the rs2853669 SNP and scrambled corresponds to the scrambled sgRNA.

| Sample Name | Total Reads | TERT CPM | VEGFA CPM | Group |
| --- | --- | --- | --- | --- |
| HEPG2_UT1 | 32,545,654 | -0.60 | 6.52 | UT |
| HEPG2_UT2 | 36,472,629 | 1.11 | 5.28 | UT |
| HEPG2_UT3 | 33,249,414 | -0.50 | 6.94 | UT |
| HEPG2_UT4 | 34,732,001 | 0.36 | 5.56 | UT |
| HEPG2_UT5 | 30,716,402 | 0.21 | 5.28 | UT |
| HEPG2_UT6 | 34,703,379 | -0.56 | 6.23 | UT |
| HEPG2_UT7 | 36,644,922 | 0.84 | 5.83 | UT |
| HEPG2_C228T-wt | 47,719,721 | -1.22 | 6.65 | C228T-wt |
| HEPG2_C228T-wt-2 | 37,078,714 | -0.54 | 6.27 | C228T-wt |
| HEPG2_C228T-wt-3 | 31,622,964 | 0.76 | 6.36 | C228T-wt |
| HEPG2_C228T-mut-1 | 74,396,857 | -7.22 | 6.71 | C228T-mut |
| HEPG2_C228T-mut-2 | 47,476,019 | -6.57 | 6.62 | C228T-mut |
| HEPG2_C228T-mut-3 | 48,369,487 | -6.60 | 6.71 | C228T-mut |
| HEPG2_scrambled | 39,351,318 | -6.30 | 6.61 | scrambled |
| HEPG2_scrambled-2 | 38,829,631 | -0.05 | 7.11 | scrambled |
| HEPG2_scrambled-3 | 30,489,705 | 0.34 | 6.79 | scrambled |
| HEPG2_scrambled_4 | 27,822,099 | -0.37 | 6.48 | scrambled |
| HEPG2_scrambled_5 | 22,150,938 | 0.51 | 5.57 | scrambled |
| A549_UT1 | 21,439,527 | -5.42 | 4.42 | UT |
| A549_UT2 | 21,601,842 | -0.39 | 4.86 | UT |
| A549_UT3 | 22,019,900 | -5.46 | 5.23 | UT |
| A549_UT4 | 22,168,859 | -5.47 | 3.94 | UT |
| A549_rs2853669-alt_1 | 20,155,551 | 0.22 | 4.81 | rs2853669-alt |
| A549_rs2853669-alt_2 | 22,502,865 | -5.49 | 4.15 | rs2853669-alt |
| A549_rs2853669-alt_3 | 24,168,716 | 0.02 | 5.15 | rs2853669-alt |
| A549_rs2853669-alt_4 | 20,756,297 | -5.38 | 4.70 | rs2853669-alt |
| A549_scrambled_1 | 21,129,944 | -5.40 | 5.16 | scrambled |
| A549_scrambled_2 | 21,524,877 | 0.13 | 4.67 | scrambled |
| A549_scrambled_3 | 19,647,280 | -5.30 | 5.48 | scrambled |
| A549_scrambled_4 | 20,281,555 | 2.42 | 4.44 | scrambled |
